# Variable parameters memory-type control charts for simultaneous monitoring of the mean and variability of multivariate multiple linear regression profiles

**DOI:** 10.1038/s41598-024-59549-8

**Published:** 2024-04-23

**Authors:** Hamed Sabahno, Marie Eriksson

**Affiliations:** Department of Statistics, Umeå School of Business, Economics and Statistics, Umeå University, Umeå, Sweden

**Keywords:** Multivariate multiple linear regression profiles, Profile monitoring, Memory-type control charts, Max-type control charts, SS-type control charts, VP adaptive control charts, Monte Carlo simulation, Healthcare, Health care, Computational science, Statistics

## Abstract

Variable parameters (VP) schemes are the most effective adaptive schemes in increasing control charts' sensitivity to detect small to moderate shift sizes. In this paper, we develop four VP adaptive memory-type control charts to monitor multivariate multiple linear regression profiles. All the proposed control charts are single-chart (single-statistic) control charts, two use a Max operator and two use an SS (squared sum) operator to create the final statistic. Moreover, two of the charts monitor the regression parameters, and the other two monitor the residuals. After developing the VP control charts, we developed a computer algorithm with which the charts' time-to-signal and run-length-based performances can be measured. Then, we perform extensive numerical analysis and simulation studies to evaluate the charts’ performance and the result shows significant improvements by using  the VP schemes. Finally, we use real data from the national quality register for stroke care in Sweden, Riksstroke, to illustrate how the proposed control charts can be implemented in practice.

## Introduction

Statistical process monitoring is a method utilized to monitor the variations in any process and to ensure the delivery of good quality outputs (products/services). Control charts are the main tools for this purpose. The first control chart was introduced by Shewhart in 1924. Since this control chart is memory-less, it is slow in detecting small and moderate shift sizes. To improve the sensitivity of the Shewhart control chart, different approaches are proposed. Two of the main approaches are using memory-type and adaptive schemes.

Although the statistic in the memory-less control charts is only related to the current sample, it is somehow related to the previous statistics as well (its value gets updated based on both current and previous samples) in the memory-type control charts. The main memory-type control charts are EWMA (exponentially weighted moving average) and CUSUM (cumulative sum) control charts.

On the other hand, in adaptive schemes, at least one of the chart’s parameters (sampling interval, sample size, and control limits) is allowed to vary from sample to sample (usually between two possible values). The main adaptive schemes are VSI (Variable Sampling Interval), VSS (Variable Sample Size), VSSI (Variable Sample Size and Sampling Interval), and VP (variable parameters). Studies such as Sabahno et al.^1^ have shown that the VP scheme, in which all the chart parameters are allowed to vary, is the best-performing scheme. For some notable works regarding adding different adaptive schemes to different control charts, we refer interested readers to Sabahno et al.^1–4^, Sabahno & Celano^5^, and Sabahno^6^.

Although adaptive schemes were initially developed to be used in memory-less control charts, studies such as Perdikis & Psarakis^7^ have shown that combining both approaches (using memory-type and adaptive control charts) further improves the chart’s sensitivity and performance. Nonetheless, there are also studies such as Amir et al.^8^ and Abbas et al.^9^ that have used auxiliary information to increase the sensitivity of memory-type control charts.

Profile monitoring is a special case of statistical process monitoring in which instead of quality characteristics, the relationship between some dependent and independent variables in the form of a regression model is monitored. Maintaining this relationship ensures the process quality, in this case. The idea of profile monitoring was raised by Kang & Albin^10^. They introduced simple linear profiles with two main applications in semiconductor and food manufacturing. They used the memory-less Hotelling´s T^2^ and the memory-type EWMA control charts. A significant development in monitoring simple linear profiles was made by Kim et al.^11^. They developed a monitoring scheme using three EWMA statistics. Other notable work has been proposed by Zou et al.^12^. They used a control chart based on a change-point model to monitor linear profiles. Some other notable works that have developed Kang & Albin^10^’s research are Yeh et al.^13^, Noorossana et al.^14^, Eyvazian et al.^15^, Hosseinifard et al.^16^, and Zou et al.^17^.

Simultaneous monitoring of the normal process parameters (the mean and variability) usually results in better overall performance. Although there are two main simultaneous monitoring schemes, one uses only one single chart and the other uses two charts, the former is preferred due to its simplicity of usage. Some notable works that have considered simultaneous monitoring of profiles parameters are Zhang et al.^18^, Eyvazian et al.^15^, Khedmati & Niaki^19^, Ghashghaei & Amiri^20,21^, Mahmood et al.^22^, Saeed et al.^23^, Ghashghaei et al.^24^, Malela-Majika et al.^25^, Abbasi et al.^26^, Sabahno & Amiri^27^, and Sherwani et al.^28^. Ghashghaei & Amiri^20^, developed two memory-type control charts by using a max-operator, namely Max-MEWMA (multivariate EWMA) and Max-MCUSUM (multivariate CUSUM) control charts for simultaneous monitoring of the mean vector and variance-covariance matrix of multivariate multiple linear profiles. Ghashghaei & Amiri^21^ did the same but this time by using an SS (squared sum) operator. They called their control charts SS-EWMA and SS-CUSUM. However, other than developing control charts to monitor the profile’s parameters (before-mentioned ones), they also developed control charts to monitor the residuals. Both these studies used simulation to compute the performance measures. Mahmood et al.^22^ developed SS and Max types EWMA control charts using three EWMA statistics and showed superior performance over using those three EWMA statistics separately (EWMA3), three separate Hotelling T^2^ charts, and EWMA-R charts. Saeed et al.^23^ developed a scheme using three progressive statistics which were monitored separately, and showed superior performance over the existing charts including EWMA3, EWMA-R, Hotelling T^2^, and a scheme with three separate Shewhart-type charts. Abbas et al.^29^ investigated the Bayesian EWMA and MEWMA control charts for monitoring of the linear profiles when the explanatory variables are random. However, in most studies like ours, the explanatory variables are assumed fixed﻿﻿.

Moreover, the following studies have considered adaptive schemes in profile monitoring. Li & Wang^30^ developed an EWMA scheme with variable sampling intervals (VSI) for monitoring linear profiles. Abdella et al.^31^ developed a Hotelling T^2^ scheme with varying sample sizes and sampling intervals (VSSI). Ershadi et al.^32^, investigated the economic-statistical design of an EWMA scheme with variable sampling interval (VSI) for linear profile monitoring. Magalhaes & Von Doellinger^33^ developed a variable sample size (VSS) χ^2^ scheme for linear profile monitoring. Kazemzadeh et al.^34^ developed the EWMA3 and MEWMA schemes with variable sample sizes. Ershadi et al.^35^ investigated the economic-statistical design of an EWMA scheme with variable sample size (VSS) for linear profile monitoring. Darbani & Shadman^36^, developed a generalized likelihood ratio control chart with variable sampling intervals for monitoring linear profiles. Yeganeh et al.^37^ developed an adaptive MEWMA control chart based on the ratio of samples. Haq^38^ developed adaptive MEWMA charts by varying the smoothing parameter for monitoring linear profiles. Sabahno & Amiri^27^ developed a VP memory-less Max-type control chart for simultaneous monitoring of the mean vector and the variance-covariance matrix in multivariate multiple linear profiles. They evaluated the chart performance using a dedicated Markov chain model. Sabahno & Amiri^39^ developed memory-less machine-learning based control charts and compared them to the best available statistical control charts for monitoring generalized linear regression profiles’ parameters in both fixed and variable parameters schemes.

According to the literature and the review paper of Perdikis & Psarakis^7^, VP adaptive schemes have not so far been developed for memory-type control charts. In this paper, we develop VP schemes for four memory-type control charts: the Max-MEWMA and Max-MCUSUM for monitoring the regression parameters (from Ghashghaei & Amiri^20^), and two SS-type control charts for monitoring the residuals (from Ghashghaei & Amiri^21^). Note that we only use Ghashghaei & Amiri^21^’s SS-type control charts for the residual, because they discovered them to be more effective than the SS-type charts to monitor the regression parameters. We also develop a computer algorithm to compute different performance measures of the developed control charts, which can be used for any other memory-type VP control chart as well. Furthermore, we use a real case to show how the proposed control charts can be implemented in practice. To do so, we use a dataset from the national Swedish Stroke Register, Riksstroke. After estimating two correlated multiple profiles, we develop and implement the proposed control charts to monitor the stroke care-related relationships.

This paper is structured as follows. Multivariate multiple linear profiles are described in Section "Multivariate multiple linear profiles". The Max-type and SS-type memory-type control charts for simultaneous monitoring of multivariate multiple linear profiles are described in Section "Max-type and SS-type memory-type control charts". In Section "Design parameters in a variable parameters scheme", VP adaptive schemes are developed for control charts described in Section "Max-type and SS-type memory-type control charts". Section "Performance measures" contains the proposed algorithm to compute the performance measures of the proposed control charts. Section "Simulation studies" contains extensive simulation studies and numerical analyses to evaluate the proposed control charts’ performance using the proposed performance measure algorithm, under different shift types and sizes. Our real case illustrative example is presented in Section "A real case". Concluding remarks and suggestions for future developments of the paper are mentioned in Section "Conclusions".

## Multivariate multiple linear profiles

For the *k*th sample of size* n*, with *p* response variables (profiles)**,** ${\mathbf{Y}}_{k}$ is a $n\times p$ matrix. ${\mathbf{Y}}_{k}$ is a linear function of some independent variables *x*, so that:

1 $${\mathbf{Y}}_{k}=\mathbf{X}\mathbf{B}+{\mathbf{\rm E}}_{k},$$

where **X** is a $n\times \left(q+1\right)$ matrix of explanatory (independent) variables,$q$ is the number of independent variables, $\mathbf{B}$ is a $\left(q+1\right)\times p$ matrix of regression parameters, and ${\mathbf{\rm E}}_{k}$ is a $n\times p$ matrix of correlated error terms ($\varepsilon )$, which follows a multivariate normal distribution (**0**,${\varvec{\Sigma}})$, where $${\varvec{\Sigma}}=\left(\begin{array}{ccc}\begin{array}{cc}{\sigma }_{11}& {\sigma }_{12}\end{array}& \cdots & {\sigma }_{1p}\\ \vdots & \ddots & \vdots \\ \begin{array}{cc}{\sigma }_{p1}& {\sigma }_{p2}\end{array}& \cdots & {\sigma }_{pp}\end{array}\right)$$, and ${\sigma }_{gh}$ denotes the covariance between the error vector terms of *g*th and the *h*th response variables at each observation.

Therefore, we can write Eq. (1) as:

$$\begin{gathered} \left( {\begin{array}{*{20}c} {\begin{array}{*{20}c} {y_{11k} } & {y_{12k} } \\ \end{array} } & \cdots & {y_{1pk} } \\ \vdots & \ddots & \vdots \\ {\begin{array}{*{20}c} {y_{n1k} } & {y_{n2k} } \\ \end{array} } & \cdots & {y_{npk} } \\ \end{array} } \right) = \left( {\begin{array}{*{20}c} {\begin{array}{*{20}c} 1 & {x_{11} } \\ \end{array} } & \cdots & {x_{1q} } \\ \vdots & \ddots & \vdots \\ {\begin{array}{*{20}c} 1 & {x_{n1} } \\ \end{array} } & \cdots & {x_{nq} } \\ \end{array} } \right)_{{n \times \left( {q + 1} \right)}} \left( {\begin{array}{*{20}c} {\begin{array}{*{20}c} {\beta_{01} } & {\beta_{02} } \\ \end{array} } & \cdots & {\beta_{0p} } \\ \vdots & \ddots & \vdots \\ {\begin{array}{*{20}c} {\beta_{q1} } & {\beta_{q2} } \\ \end{array} } & \cdots & {\beta_{qp} } \\ \end{array} } \right)_{{\left( {q + 1} \right) \times p}} + \\ \left( {\begin{array}{*{20}c} {\begin{array}{*{20}c} {\varepsilon_{11k} } & {\varepsilon_{12k} } \\ \end{array} } & \cdots & {\varepsilon_{1pk} } \\ \vdots & \ddots & \vdots \\ {\begin{array}{*{20}c} {\varepsilon_{n1k} } & {\varepsilon_{n2k} } \\ \end{array} } & \cdots & {\varepsilon_{npk} } \\ \end{array} } \right)_{n \times p} . \\ \end{gathered}$$

## Max-type and SS-type memory-type control charts

In this section, we describe two memory-type Max-type control charts, namely Max-MEWMA, and Max-MCUSUM charts, which form a single statistic by taking the maximum value between the absolute values of two statistics (one for the process mean vector and the other one for the process variability). We also describe two SS-type memory type control charts, namely SS-EWMAe and SS-CUSUMe charts, which form a single statistic by adding the squared values of two statistics (again, one for the process mean vector and the other one for the process variability). As previously mentioned in the Introduction section, these Max-type and SS-type control charts are proposed by Ghashghaei & Amiri^20^ and Ghashghaei & Amiri^21^, respectively.

### Memory-type control charts using a Max operator

In this section, we describe two memory type Max-type control charts for regression parameters, namely Max-MEWMA and Max-MCUSUM charts, which were introduced by Ghashghaei & Amiri^20^.

#### Max-MEWMA control chart

In this section, we detail a single control chart for simultaneously monitoring of the process mean vector and variability ($\mathbf{B}$ and ${\varvec{\Sigma}}$ matrices), by assuming that their values are known.

First, we need to develop a statistic to represent the process mean vector. To monitor the process mean, the Hotelling’s ${T}_{k}^{2}$ statistic can be used to monitor the changes in the $\mathbf{B}$ matrix. The sample estimate of the $\mathbf{B}$ matrix (${\widehat{\mathbf{\rm B}}}_{k}$) is computed as:

2 $${\widehat{\mathbf{\rm B}}}_{k}={\left[{\mathbf{X}}^{T}\mathbf{X}\right]}^{-1}{\mathbf{X}}^{{\text{T}}}{\mathbf{Y}}_{k}.$$

By changing ${\widehat{\mathbf{\rm B}}}_{k}$ into a *p*(*q*+1)$\times 1$ vector, we have:

$${\widehat{{\varvec{\beta}}}}_{k}={\left({\widehat{\beta }}_{01k},{\widehat{\beta }}_{11k},\dots ,{\widehat{\beta }}_{q1k},\dots \dots ,{\widehat{\beta }}_{0pk},{\widehat{\beta }}_{1pk},\dots ,{\widehat{\beta }}_{qpk}\right)}^{T}.$$

Next, we need to compute its average and variance-covariance matrix. For an in-control process, and since we assume that the parameters’ values are known, the expected value of ${\widehat{\boldsymbol{\rm B}}}_{k}$ is equal to ${\varvec{B}}$. Therefore, we have:

$${\varvec{\beta}}=E({\widehat{{\varvec{\beta}}}}_{k})={\left({\beta }_{01},{\beta }_{11},\dots ,{\beta }_{q1},\dots \dots ,{\beta }_{0p},{\beta }_{1p},\dots ,{\beta }_{qp}\right)}^{T}.$$

For its variance-covariance matrix, we have:

$${{\varvec{\Sigma}}}_{{\widehat{{\varvec{\beta}}}}_{k}}={\left(\begin{array}{ccc}\begin{array}{cc}{{\varvec{\Sigma}}}_{11}& {{\varvec{\Sigma}}}_{12}\end{array}& \cdots & {{\varvec{\Sigma}}}_{1p}\\ \vdots & \ddots & \vdots \\ \begin{array}{cc}{{\varvec{\Sigma}}}_{p1}& {{\varvec{\Sigma}}}_{p2}\end{array}& \cdots & {{\varvec{\Sigma}}}_{pp}\end{array}\right)}_{p\left(q+1\right)\times p\left(q+1\right)},$$

${\boldsymbol{\Sigma }}_{gh}$ is a $\left(q+1\right)\times \left(q+1\right)$ matrix equal to ${\left[{{\varvec{X}}}^{T}{\varvec{X}}\right]}^{-1}{\sigma }_{gh}$.

Next, we define:

3 $${{\varvec{z}}}_{k}=\lambda \left({\widehat{{\varvec{\beta}}}}_{k}-{{\varvec{\beta}}}_{k}\right)+\left(1-\lambda \right){{\varvec{z}}}_{k-1},$$

where ${{\varvec{z}}}_{0}$ is the starting point and is equal to zero, $\lambda$ is the smoothing parameter and its value can vary between 0 and 1. However, most commonly, $\lambda =0.2$ is used.

Then, the following statistic is defined for monitoring the process mean vector:

4 $${C}_{k}={\boldsymbol{\Phi }}^{-1}\left[{{\varvec{H}}}_{\left(q+1\right)p}\left\{\frac{2-\lambda }{\lambda }{{\varvec{z}}}_{k}^{T}{\Sigma }_{\widehat{{\varvec{\beta}}}}^{-1}{{\varvec{z}}}_{k}\right\}\right],$$

where ${{\varvec{H}}}_{\left(q+1\right)p}\left(.\right)$ is the chi-square cumulative distribution function with $\left(q+1\right)p$ degrees of freedom, and $\Phi (.)$ is the standard normal cumulative distribution function.

To construct the statistic for monitoring the process variability, first, we define ${W}_{k}$ as:

5 $${W}_{k}={\sum }_{i=1}^{n}\left({y}_{ik}-{x}_{i}\mathbf{B}\right){{\varvec{\Sigma}}}^{-1}{({y}_{ik}-{x}_{i}\mathbf{B})}^{T}.$$

So that ${W}_{k}$ has a chi-square distribution with *np* degrees of freedom. Then, we define:

6 $${g}_{k}=\left(1-\lambda \right){g}_{k-1}+\lambda {\boldsymbol{\Phi }}^{-1}\left[{{\varvec{H}}}_{np}\left\{{W}_{k}\right\}\right],$$

where ${g}_{0}$ is the starting point and is equal to zero. The statistic for monitoring the variability is defined as:

7 $${S}_{k}=\sqrt{\frac{2-\lambda }{\lambda }}{g}_{k},$$

Finally, the $M{E}_{k}$ statistic is formed by combining ${C}_{k}$ and ${S}_{k}$:

8 $$M{E}_{k}=max\left\{\left|{C}_{k}\right|,\left|{S}_{k}\right|\right\}.$$

Since this statistic only generates positive values, we only need an upper control limit (*UCL*) for this control chart, and its value is obtained using simulation to achieve any desired ARL performance.

#### Max-MCUSUM control chart

The statistic for monitoring the process mean vector for the Max-MCUSUM control chart is defined as:

9 $${U}_{k}=max\left\{0,{U}_{k-1}+{Z}_{k}-0.5D\right\},$$

where ${Z}_{k}=a{\left({\widehat{{\varvec{\beta}}}}_{k}-{{\varvec{\beta}}}_{g}\right)}^{T}$, $a={\frac{\left({{\varvec{\beta}}}_{b}-{{\varvec{\beta}}}_{g}\right){\boldsymbol{\Sigma }}_{\widehat{{\varvec{\beta}}}}^{-1}}{\sqrt{\left({{\varvec{\beta}}}_{b}-{{\varvec{\beta}}}_{g}\right){\boldsymbol{\Sigma }}_{\widehat{{\varvec{\beta}}}}^{-1}{\left({{\varvec{\beta}}}_{b}-{{\varvec{\beta}}}_{g}\right)}^{T}}}}$, ${{\varvec{\beta}}}_{g}$ is the good ${\varvec{\beta}}$ (in-control mean vector)***,*** ${{\varvec{\beta}}}_{b}$ is the bad ${\varvec{\beta}}$ (out-of-control mean vector), D=$\sqrt{\left({{\varvec{\beta}}}_{b}-{{\varvec{\beta}}}_{g}\right){\Sigma }_{\widehat{{\varvec{\beta}}}}^{-1}{\left({{\varvec{\beta}}}_{b}-{{\varvec{\beta}}}_{g}\right)}^{T}}$ and ${U}_{0}=0$.

The statistic for monitoring the process variability is:

10 $${L}_{k}=max\left\{0,{L}_{k-1}+\left({\widehat{{\varvec{\beta}}}}_{k}-{{\varvec{\beta}}}_{g}\right){\boldsymbol{\Sigma }}_{\widehat{{\varvec{\beta}}}}^{-1}{\left({\widehat{{\varvec{\beta}}}}_{k}-{{\varvec{\beta}}}_{g}\right)}^{T}-v\right\},$$

where $v={\text{log}}\left(\tau \right){\left(\frac{\tau }{\tau -1}\right)}$, $\tau$ is the multiplier with which the variance-covariance matrix shifts ($\boldsymbol{\Sigma }\to \tau \boldsymbol{\Sigma })$, and similar to Ghashghaei and Amiri^20^ we assumed $\tau$ =1.1 and ${L}_{0}=0$.

Finally, $M{C}_{k}$ is formed by combining ${U}_{k}$ and ${L}_{k}$, as:

11 $$M{C}_{k}=max\left\{{U}_{k},{L}_{k}\right\}.$$

Again, since this statistic only generates positive values, we only need a *UCL* for this control chart, and its value is obtained using simulation to achieve any desired ARL performance.

### Memory-type control charts using a SS operator

In this section, we describe the SS-type control charts for the residuals introduced by Ghashghaei & Amiri^21^. They developed these kinds of control charts to monitor both regression parameters and residuals (four control charts). However, we only use the ones for monitoring the residuals, mostly because we already have our Max-type ones for monitoring the regression parameters, and also, they concluded that in most situations their residual monitoring charts perform better than the other ones.

#### SS-EWMAe control chart

To compute the statistic for monitoring the mean vector ($${P}_{k})$$, we first define:

12 $${T}_{k}={\boldsymbol{\Phi }}^{-1}\left[{{\varvec{H}}}_{p}\left\{{{\varvec{z}}}_{k}^{T}{\Sigma }_{\widehat{{\varvec{\beta}}}}^{-1}{{\varvec{z}}}_{k}\right\}\right],$$

where ${{\varvec{H}}}_{p}\left(.\right)$ is the chi-square cumulative distribution function with $p$ degrees of freedom, $\Phi (.)$ is the standard normal cumulative distribution function, ${\varvec{z}}_{k} = \lambda \overline{e}_{k} + \left( {1 - \lambda } \right){\varvec{z}}_{k - 1}$, ${{\varvec{z}}}_{0}=0$**,** $\overline{e}_{k} = \left( {\overline{e}_{1k} ,\overline{e}_{2k} , \ldots ,\overline{e}_{pk} } \right)$ is the average residual vector in the sample *k,* and $\lambda$ is the smoothing parameter.

Then, we have:

13 $${P}_{k}=\lambda {T}_{k}+\left(1-\lambda \right){P}_{k-1},$$

where ${P}_{0}$ is equal to zero.

To compute the statistic for monitoring the variability, we first have:

14 $${F}_{k}={\boldsymbol{\Phi }}^{-1}\left[{{\varvec{H}}}_{np}\left\{{f}_{k}\right\}\right],$$

where ${f}_{k}={\sum }_{i=1}^{n}\left({e}_{ik}\right){{\varvec{\Sigma}}}^{-1}({e}_{ik}$)^*T*^, and ${\varvec{\Sigma}}$ is the variance-covariance matrix of the error terms.

Then we have:

15 $${V}_{k}=\lambda {F}_{k}+\left(1-\lambda \right){V}_{k-1},$$

where $${V}_{0}=0$$.

Finally, the SS-type statistic in the EWMAe scheme is defined as:

16 $$EW{e}_{k}={P}_{k}^{2}+{V}_{k}^{2}.$$

The same as in the case of Max-type control charts, since this statistic only generates positive values, we only need a UCL for this control chart, and its value is obtained using simulation to achieve any desired ARL performance.

#### SS-CUSUMe control chart

The statistic for monitoring the mean vector in this scheme is:

17 $${M}_{k}=max\left\{{D}_{k}^{-},{D}_{k}^{+}\right\},$$

where ${D}_{k}^{-}=max\left\{0,-{T}_{k}-{k}_{1}+{D}_{k-1}^{-}\right\}$, ${D}_{k}^{+}=max\left\{0,{T}_{k}-{k}_{1}+{D}_{k-1}^{+}\right\}$, ${D}_{0}^{-}=0, {D}_{0}^{+}=0,$ ${k}_{1}$ is the reference value, and ${T}_{k}$ was defined in the previous chart.

Similarly, the statistic for monitoring the process variability is:

18 $${N}_{k}=max\left\{{B}_{k}^{-},{B}_{k}^{+}\right\},$$

where ${B}_{k}^{-}=max\left\{0,-{F}_{k}-{k}_{2}+{B}_{k-1}^{-}\right\}$, ${B}_{k}^{+}=max\left\{0,{F}_{k}-{k}_{2}+{B}_{k-1}^{+}\right\}$, ${B}_{0}^{-}=0, {B}_{0}^{+}=0,$ ${k}_{2}$ is the reference value, and ${F}_{k}$ was defined in the previous chart.

Finally, the SS-type statistic in the CUSUMe scheme is defined as:

19 $$CU{e}_{k}={M}_{k}^{2}+{N}_{k}^{2}.$$

Note that following Ghashghaei & Amiri^21^, in this paper we choose ${k}_{1}=1$ and ${k}_{2}=1.5$.

## Design parameters in a variable parameters scheme

The adaptive scheme in which all the control chart (design) parameters are allowed to vary from sample to sample, is called a VP (Variable Parameters) scheme. In this paper, we consider two types of sample sizes with ${n}_{1}$<${n}_{2}$, two types of sampling intervals with ${t}_{2}$< ${t}_{1}$, and two types of Type-I error probabilities with ${\alpha }_{1}$<${\alpha }_{2}$. In addition to these parameters, we have to define two upper control limits $UC{L}_{1}$ and $UC{L}_{2}$ with $UC{L}_{2}<UC{L}_{1}$ as well as two upper warning limits $UW{L}_{1}$ and $UW{L}_{2}$, satisfying $UW{L}_{1}<UC{L}_{1}$ and $UW{L}_{2}<UC{L}_{2}$.

In a VP scheme, we should have the following three constraints (each one is related to one design parameter) satisfied:

20 $$E(n)={n}_{1}{P}_{0}+{n}_{2}(1-{P}_{0}),$$

21 $$E(t)={t}_{1}{P}_{0}+{t}_{2}(1-{P}_{0}),$$

22 $$E(\alpha )={\alpha }_{1}{P}_{0}+{\alpha }_{2}(1-{P}_{0}).$$

By solving Eqs. (20)–(22) together, ${P}_{0}$, ${t}_{1}$ and ${\alpha }_{2}$ are obtained as:

23 $${P}_{0}=\frac{E(n)-{n}_{2}}{{n}_{1}-{n}_{2}},$$

24 $${t}_{1}=\frac{E(t)({n}_{1}-{n}_{2})-{t}_{2}({n}_{1}-E(n))}{E(n)-{n}_{2}},$$

25 $${\alpha }_{2}=\frac{E(\alpha )({n}_{1}-{n}_{2})-{\alpha }_{1}(E(n)-{n}_{2})}{{n}_{1}-E(n)}.$$

Note that ${P}_{0}$ is the conditional probability of being in the safe zone while the process is in-control. After determining the values of the *UCL*s and *UWL*s, which we will later show how to determine by introducing algorithms 1 and 2, we use the following sampling strategy in a VP scheme:

- If at sample *k*, the statistic’s output $\in \left[0,UW{L}_{(k)}\right]$, then the process is declared as being in-control and the parameters for the next sample must be ${n}_{1},{t}_{1},UC{L}_{1},UW{L}_{1}$.
- If at sample *k*, the statistic’s output $\in \left(UW{L}_{(k)},UC{L}_{(k)}\right]$, then the process is also declared as being in-control, but the parameters for the next sample must be ${n}_{2},{t}_{2},UC{L}_{2},UW{L}_{2}$.
- If at sample *k*, the statistic’s output $\in \left(UC{L}_{(k)},\infty \right)$, then the process is declared as being out-of-control and the corrective actions might be required,

where $UC{L}_{(k)}\in \left\{UC{L}_{1},UC{L}_{2}\right\}$ and $UW{L}_{(k)}\in \left\{UW{L}_{1},UW{L}_{2}\right\}$ are the upper control and warning limits used for sample *k*=1,2,..., respectively.

For determining the *UCL* values, we assume that we have an FP scheme and set the values of each *UCL* separately (*UCL* for an FP control chart, and *UCL*_*1*_ & *UCL*_*2*_ for a VP control chart). We use the following algorithm for determining the values of *UCL*s. First note that in all the following algorithms, it is assumed that the average sampling interval is equal to one time unit, which results in ARL=ATS. Otherwise, it would have been ATS=*t* $\times$ ARL. Also, all the algorithms in this section should be run in an in-control state. In fact, obtaining the values of all the control chart parameters should be performed while the process is in-control.

### Algorithm 1: Adjusting the UCL values

Step 1-Choose a value for $\alpha$ (the probability of Type-I error) and the sample size *n*.

Note that for the FP schemes there is only one $\alpha$ and one *n*. However, for the VP schemes we have two $\alpha$s and two *n*s, therefore we set *UCL*_*1*_ by using ${\alpha }_{1}$ and ${n}_{1}$, and *UCL*_*2*_ by using ${\alpha }_{2}$ and ${n}_{2}$.

Step 2-Choose a statistic (*ME*, *MC*, *EWe*, or *CUe*).

Step 3-Obtain the initial value for the *UCL* by generating and increasingly sorting 10000 in-control samples by using the statistic and choosing the [10000(1-$\alpha$)]^th^ value in the range.

Step 4-Run 10000 simulations and adjust the *UCL* so that you get ARL=$\frac{1}{\alpha }$.

After determining the value of the *UCL*s, we should obtain the values of *UWL*s for the VP scheme (remember that the FP scheme has no *UWL*). For obtaining the *UWL* values, we can assume that we only have a variable sampling interval (VSI) scheme and compute each *UWL* separately with its corresponding parameters using the following algorithm (as is supposed to be done in a VSI scheme).

### Algorithm 2: Adjusting the UWL values

Step 1-Choose the values of $\alpha$, *t*_2_,* E*(*t*), the corresponding *UCL* value obtained via the previous algorithm, and the corresponding sample size (${n}_{1}$&*UCL*_*1*_ if $\alpha$ =${\alpha }_{1}$, and ${n}_{2}$&*UCL*_*2*_ if $\alpha$ =${\alpha }_{2}$).

Step 2-Compute ${P}_{0}$ using Eq. (23) and *t*_1_ using Eq. (24).

Step 3-Choose the same statistic used for determining the corresponding *UCL* value.

Step 4-Run 10000 simulations and adjust each *UWL* with its corresponding *UCL* so that you get ${P}_{0}$ equal to the value you obtained in Step 3 and at the same time get ARL=$\frac{1}{\alpha }$ and ATS=* E*(*t*)ARL=$E(t)\frac{1}{\alpha }$ (note that if *E*(*t*)=1, as in our case, then ARL= ATS=$\frac{1}{\alpha }$).

## Performance measures

The Run length and time to signal based measures are the two most important control charts’ performance measures. In an FP scheme, computing the average run length based measures are enough, considering one can multiply the average run length by the sampling interval to obtain the average time to signal. However, in a VP scheme, the average time to signal should be computed separately. Both average and standard deviation of run length (ARL and SDRL) as well as time to signal (ATS and SDTS), are important to consider. Although the SDRL and SDTS are always expected to be low, the ARL and ATS are expected to be as high as possible when the process is in-control and as low as possible when the process is out-of-control.

To compute the performance measures for an FP scheme, algorithm 1 can still be used with the only difference that here we have obtained the *UCL* value, and we are now only interested in computing the values of the ARL and SDTS in an out-of-control situation.

To compute the performance measures for a VP control chart, the following computer algorithm is developed and can be used. Note that, to reduce the paper size, we only present the algorithm for the case of the Max-MEWMA control chart, but it can easily be modified for the other proposed control charts as well.

Algorithm 3 Computing the performance measures in a memory-type VP scheme.
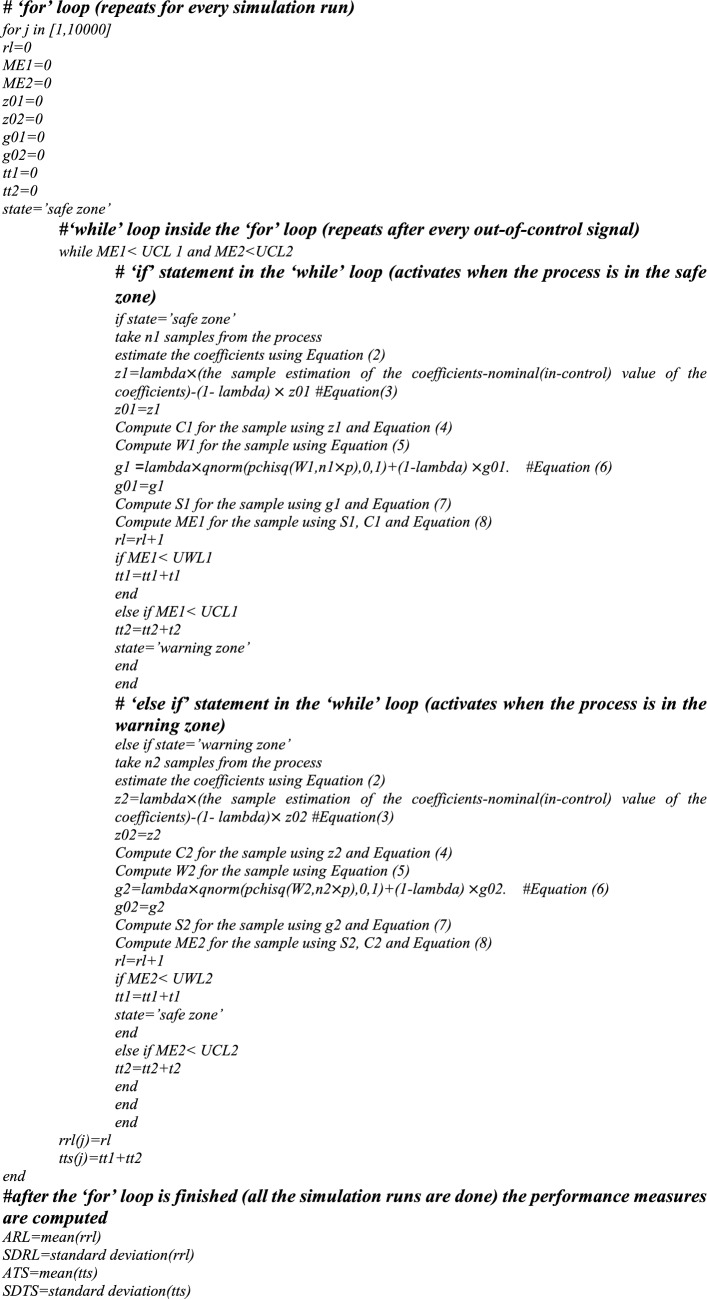


As can be seen in the above-mentioned algorithm, the final statistic is differentiated with ‘*ME*1’ and ‘*ME*2’ depending on their zone (safe or warning zone). This happens in all the control charts whether they are memory-type or memory-less. However, if we have a memory-type control chart, the memory-type statistics (g and z statistics in Max-MEWMA) should also be divided into two categories as well and the previous statistic values obtained in region 1(2) cannot be used for the next sampling in region 2(1). In other words, region 1 values should only be used when we are in region 1 and the same applies to region 2 values. Otherwise, this will significantly increase the false alarm rate.

## Simulation studies

In this section, we perform numerical analyses and simulation studies to evaluate and compare our developed adaptive control charts to one another in adaptive and one-adaptive conditions. We﻿ evaluate the performance of the proposed control charts under different shift scenarios and in different dimensions.

Although we report the values of the ATS, SDTS, ARL, and SDRL in the following tables, the comparisons are mainly made using the ATS values. All the simulation environments and the chosen values for the process and chart parameters as well as the shift sizes are the same as in Sabahno & Amiri^27^. The in-control ARL and ATS for all the considered control charts are set to 200 runs and 200 *hrs* (α = 0.005), respectively. The analysis is conducted for the case of two and six response variables (*p*=2 and 6), i.e. two and six multiple linear profiles. The following multiple regression models are used for the case of *p*=2: ${y}_{1}=3+2{x}_{1}+{x}_{2}+{\varepsilon }_{1}$ and ${y}_{2}=2+{x}_{1}+{x}_{2}+{\varepsilon }_{2}.$

The error’s variance-covariance matrix for this case is assumed to have the following elements: $$\boldsymbol{\Sigma }=\left[\begin{array}{cc}{\sigma }_{1}^{2}& \rho {\sigma }_{1}{\sigma }_{2}\\ \rho {\sigma }_{1}{\sigma }_{2}& {\sigma }_{1}^{2}\end{array}\right]$$, where ${\sigma }_{1}$ and ${\sigma }_{2}$ are the standard deviations of the first and second profiles, respectively, and $\rho$ is their correlation. For its in-control value, we have: $${\boldsymbol{\Sigma }}_{0}=\left[\begin{array}{cc}1& 0.5\\ 0.5& 1\end{array}\right].$$

The sample size for the non-adaptive (FP) scheme is 4 and the sampling interval is 1*hr*. For the adaptive VP control chart, in which we need two values for each chart parameter, the following parameters’ values are chosen: ${n}_{1}=4$, ${n}_{2}=8$, $E\left(n\right)=6$, $E\left(\alpha \right)=0.005$, ${\alpha }_{1}=0.004$,$E\left(t\right)=1$ *hr,*and ${t}_{2}=0.1$ *hr.* ${t}_{1}$ is computed by using Eq. (24) as 1.9 *hrs*, and the upper control and warning limits are computed using the algorithms outlined in Section "Design parameters in a variable parameters scheme".

For each sample size, a set of explanatory variables is required. The **X** matrix for $n=4$ is assumed to be $$\mathbf{X}=\left(\begin{array}{ccc}1& 2& 1\\ 1& 4& 2\\ \begin{array}{c}1\\ 1\end{array}& \begin{array}{c}6\\ 8\end{array}& \begin{array}{c}3\\ 2\end{array}\end{array}\right)$$ and for the sample size 8 (only needed for the VP chart) it is considered $$\mathbf{X}=\left(\begin{array}{ccc}1& 2& 1\\ 1& 4& 2\\ \begin{array}{c}1\\ \begin{array}{c}1\\ 1\\ \begin{array}{c}1\\ 1\\ 1\end{array}\end{array}\end{array}& \begin{array}{c}6\\ \begin{array}{c}8\\ 9\\ \begin{array}{c}10\\ 9\\ 11\end{array}\end{array}\end{array}& \begin{array}{c}3\\ \begin{array}{c}2\\ 3\\ \begin{array}{c}1\\ 2\\ 1\end{array}\end{array}\end{array}\end{array}\right)$$.

We also assume that each element in the error’s variance-covariance matrix shifts with the same multiplier $(\tau ).$ In addition, the correlations between the responses in this section are assumed to be equal (regarding the *p*=6 case) and are fixed at $\rho =0.5$.

The results for the *p*=2 case are presented in Table 1 (which contains separate and simultaneous shifts in the variability and the intercepts), Table 2 (which contains separate and simultaneous shifts in the variability and the first slopes), and Table 3 (which contains separate and simultaneous shifts in the variability and the second slopes).

**Table 1:** ATS=ARL, SDTS=SDRL for FP and ATS, SDTS (ARL, SDRL) for VP schemes for shifts in the intercept vector (${{\varvec{\beta}}}_{0}$) and the error variation ($\tau$), when *p*=2 and *q*=2.

	***Shift in*** ${\varvec \beta }_{0}=\left({\beta }_{01}, {\beta }_{02}\right)$						
$\tau$	Control chart	(0, 0)	(0.1, 0.1)	(0.2, 0)	(0.2, 0.2)	(0.5, 0.5)	(1, 1)
FP scheme							
1	MAX-MEWMA	200, 200	181.43, 180.85	111.62, 108.78	112.51, 110.08	15.33, 9.94	4.68, 1.5
	MAX-MCUSUM	200, 195	53.86, 46.46	53.26, 43.31	22.81, 15.27	7.48, 2.97	3.58, 0.95
	SS-EWMAe	200, 190	99.3, 89.97	38.08, 30.1	38.78, 30.89	8.82, 3.62	4.19, 0.97
	SS-CUSUMe	200, 200	99.59, 91.57	38.03, 30.92	38.07, 32.07	8.7, 3.54	4.23, 0.92
1.1	MAX-MEWMA	108.71, 105.42	97.49, 95.82	63.6, 59.05	65.36, 60.28	13.31, 8.21	4.55, 1.52
	MAX-MCUSUM	159.94, 152.48	48.49, 42.04	47.49, 40.18	22.29, 15.48	7.54, 3.19	3.57, 1.01
	SS-EWMAe	143, 134.89	77.17, 72.95	32.72, 26.11	32.84, 26.98	8.55, 3.54	4.13, 1.02
	SS-CUSUMe	148.21, 145.14	83.89, 73.29	34.6, 28.17	34.35, 28.07	8.61, 3.63	4.19, 1
1.3	MAX-MEWMA	26.19, 22.23	24.63, 20.12	21.44, 17.28	21.66, 17.14	10.02, 5.92	4.23, 1.51
	MAX-MCUSUM	106.76, 100.12	40.1, 33.42	41.04, 33.32	20.92, 14.24	7.42, 3.26	3.58, 1.09
	SS-EWMAe	50.95, 45.33	37.88, 32.28	23.17, 17.13	23.15, 16.85	8.05, 3.63	4, 1.05
	SS-CUSUMe	89.39, 81.7	58.23, 53.27	29.39, 23.88	28.67, 22.69	8.59, 4.08	4.13, 1.07
2	MAX-MEWMA	5.18, 2.68	5.14, 2.56	5.09, 2.61	5.08, 2.59	4.39, 2.03	3.12, 1.22
	MAX-MCUSUM	14.3, 9.32	12.95, 8.2	12.38, 7.64	11.03, 6.51	6.63, 3.15	3.53, 1.23
	SS-EWMAe	7.91, 4.12	7.83, 4.03	7.38, 3.72	7.37, 3.75	5.45, 2.33	3.48, 1.1
	SS-CUSUMe	14.17, 10.84	13.6, 10.03	11.74, 8.08	11.53, 7.9	6.76, 3.49	3.67, 1.26
VP scheme							
1	MAX-MEWMA	200, 205 (200, 205)	160.46, 166.95 (164.77, 168.56)	65.49, 62.3 (74.51, 68.26)	64.23, 61.05 (73.39, 67.15)	9.093, 5.5 (12.11, 5.06)	3.66, 2.1 (5.1, 1.41)
	MAX-MCUSUM	200, 200 (200, 195)	37.28, 30.53 (48.99, 37.93)	35.62, 29.11 (48.13, 36.74)	12.59, 9.69 (19.89, 12.57)	3.22, 2.25 (6, 2.21)	1.42, 0.68 (3.04, 0.68)
	SS-EWMAe	200, 180 (200, 180)	86.96, 71.93 (88.78, 72.16)	31.6, 18.89 (32.31, 18.24)	30.89, 18.45 (31.82, 17.64)	11.52, 4.18 (10.92, 3.07)	5.1, 1.94 (5.58, 1.27)
	SS-CUSUMe	200, 180 (200, 180)	79.46, 72.87 (83.41, 72.34)	26.52, 19.67 (30.04, 19.15)	26.94, 19.54 (30.41, 19)	7.914, 4.1 (9.75, 2.95)	3.38, 1.71 (5.05, 1.14)
1.1	MAX-MEWMA	82, 81.93 (92.22, 88.33)	66.44, 61.71 (76.48, 68.01)	35.6, 31.58 (44.68, 37.03)	36.01, 31.85 (45.2, 37.03)	7.95, 5.13 (11.11, 4.72)	3.4, 2 (4.91, 1.36)
	MAX-MCUSUM	135.03, 126.13 (145.63, 132.93)	31.71, 27 (43.79, 35.07)	29.54, 24.34 (42.01, 32.42)	11.78, 8.94 (18.81, 12.01)	3.17, 2.28 (5.91, 2.27)	1.43, 0.69 (3.03, 0.69)
	SS-EWMAe	118.4, 104.8 (129.91, 114.94)	57.3, 46.63 (62.8, 50.74)	26.86, 16.01 (28.73, 15.88)	26.67, 15.9 (28.5, 15.81)	10.87, 4.34 (10.59, 3.21)	4.82, 1.99 (5.43, 1.28)
	SS-CUSUMe	142.03, 132.15 (147.66, 133.7)	64.17, 55.47 (69.71, 56.27)	23.97, 17.09 (28.15, 17.26)	24.57, 17.84 (28.32, 17.49)	7.62, 4.12 (9.58, 3.01)	3.2, 1.76 (4.92, 1.2)
1.3	MAX-MEWMA	12.14, 10.28 (19.32, 13.66)	11.56, 9.63 (18.09, 12.24)	10.16, 8.41 (16.21, 10.48)	9.8, 7.81 (15.75, 9.9)	5.55, 3.82 (8.72, 3.66)	2.87, 1.78 (4.51, 1.29)
	MAX-MCUSUM	57.59, 52.95 (73.54, 65.29)	20.61, 17.03 (31.35, 23.98)	19.14, 16.3 (30.2, 23.6)	9.78, 7.56 (16.66, 11)	3.06, 2.19 (5.79, 2.31)	1.46, 0.74 (3.03, 0.77)
	SS-EWMAe	23.95, 16.3 (35.89, 24.52)	21.11, 13.2 (29.41, 18)	15.88, 8.7 (19.77, 9.78)	16.01, 8.95 (19.9, 9.78)	8.88, 4 (9.46, 2.94)	4.33, 1.98 (5.09, 1.26)
	SS-CUSUMe	65.35, 59.6 (81.19, 71.57)	38.43, 32.75 (49.19, 39.18)	17.85, 12.96 (23.87, 14.69)	18.09, 13.09 (24.15, 14.9)	6.41, 3.83 (8.88, 2.99)	2.93, 1.66 (4.69, 1.18)
2	MAX-MEWMA	2.58, 1.77 (4.79, 1.78)	2.59, 1.83 (4.78, 1.77)	2.52, 1.77 (4.67, 1.71)	2.54, 1.76 (4.71, 1.68)	2.35, 1.57 (4.27, 1.48)	1.87, 1.14 (3.33, 0.99)
	MAX-MCUSUM	5.34, 4.54 (9.47, 6.53)	4.58, 3.69 (8.53, 5.57)	4.33, 3.49 (8.18, 5.24)	3.92, 3.11 (7.65, 4.69)	2.43, 1.68 (4.7, 2.07)	1.46, 0.78 (2.81, 0.84)
	SS-EWMAe	5.22, 2.78 (7.47, 2.52)	5.22, 2.75 (7.37, 2.44)	5.06, 2.68 (7.14, 2.35)	5.05, 2.65 (7.11, 2.36)	4.38, 2.32 (5.91, 1.84)	2.9, 1.65 (4.12, 1.11)
	SS-CUSUMe	4, 3.16 (8.06, 4.18)	3.92, 3.02 (7.89, 3.81)	3.76, 2.81 (7.43, 3.47)	3.67, 2.79 (7.34, 3.46)	2.98, 2.12 (5.6, 2.06)	2.01, 1.26 (3.75, 1.09)

**Table 2:** ATS=ARL, SDTS=SDRL for FP and ATS, SDTS (ARL, SDRL) for VP schemes for shifts in the first slope vector (${{\varvec{\beta}}}_{1}$) and the error variation ($\tau$Æ), when *p*=2 and *q*=2.

	***Shift in*** ${{\varvec{\beta}}}_{1}=\left({\beta }_{11},{\beta }_{12}\right)$						
$\tau$	Control chart	(0, 0)	(0.02, 0.02)	(0.05, 0)	(0.05, 0.05)	(0.1, 0.1)	(0.2, 0.2)
FP scheme							
1	MAX-MEWMA	200, 200	173.1, 170.96	61.19, 57.67	63.91, 59.18	12.61, 7.34	4.13, 1.22
	MAX-MCUSUM	200, 195	45.7, 35.91	35.4, 27.19	14.73, 8.15	6.38, 2.34	3.09, 0.76
	SS-EWMAe	200, 190	103.66, 93.87	25.92, 18.27	25.78, 18.67	8.79, 3.59	4.12, 0.96
	SS-CUSUMe	200, 200	100.58, 94.29	25.52, 18.94	25.68, 18.56	8.73, 3.44	4.21, 0.96
1.1	MAX-MEWMA	108.71, 105.42	93.83, 92.41	41.97, 36.97	42.15, 36.15	11.3, 6.62	4.08, 1.28
	MAX-MCUSUM	159.94, 152.48	42.33, 34.54	33.09, 25.37	14.31, 8.34	6.35, 2.49	3.12, 0.82
	SS-EWMAe	143, 134.89	77.07, 67.98	23.37, 16.54	23.18, 16.55	8.53, 3.56	4.03, 1.01
	SS-CUSUMe	148.21, 145.14	82.06, 74.78	24.45, 17.51	24.41, 17.5	8.65, 3.6	4.18, 1.02
1.3	MAX-MEWMA	26.19, 22.23	25.05, 21.39	18.06, 13.37	18.03, 13.7	9.03, 5.08	3.82, 1.27
	MAX-MCUSUM	106.76, 100.12	37.8, 31.47	29.04, 22.18	13.84, 8.35	6.41, 2.63	3.1, 0.88
	SS-EWMAe	50.95, 45.33	38.28, 32.12	17.81, 12.53	18.23, 12.48	7.97, 3.54	3.88, 1.07
	SS-CUSUMe	89.39, 81.7	58.53, 52.15	21.38, 15.86	21.37, 15.54	8.48, 3.91	4.07, 1.1
2	MAX-MEWMA	5.18, 2.68	5.15, 2.63	4.96, 2.52	4.97, 2.46	4.31, 2.01	2.91, 1.08
	MAX-MCUSUM	14.3, 9.32	12.75, 8.03	11.65, 7.31	9.61, 5.46	5.88, 2.66	3.13, 1.03
	SS-EWMAe	7.91, 4.12	7.65, 3.98	6.97, 3.29	7.1, 3.54	5.38, 2.19	3.32, 1.02
	SS-CUSUMe	14.17, 10.84	13.54, 10.02	10.73, 7.14	10.59, 7.03	6.62, 3.35	3.63, 1.31
VP scheme							
1	MAX-MEWMA	200, 205 (200, 205)	111.68, 113.05 (118.81, 117)	14.64, 10.64 (18.82, 10.69)	14.42, 10.7 (18.64, 10.56)	5.74, 3.91 (7.16, 2.57)	2.36, 1.48 (3.52, 0.86)
	MAX-MCUSUM	200, 200 (200, 195)	19.61, 15.74 (27.68, 19.46)	12.63, 9.83 (19.33, 12.07)	5.2, 3.97 (8.04, 3.45)	2.28, 1.62 (3.89, 1.12)	1.23, 0.46 (2.21, 0.43)
	SS-EWMAe	200, 180 (200, 180)	54.33, 41.09 (55.18, 39.98)	18.7, 9.32 (17.45, 6.48)	18.75, 9.47 (17.46, 6.54)	9.77, 4.04 (9.13, 2.46)	3.7, 1.74 (4.36, 0.98)
	SS-CUSUMe	200, 180 (200, 180)	49.57, 41.26 (53.01, 40.27)	12.92, 8.55 (14.67, 6.38)	13.06, 8.43 (14.74, 6.27)	6.06, 3.64 (7.34, 2.23)	2.49, 1.4 (3.68, 0.87)
1.1	MAX-MEWMA	82, 81.93 (92.22, 88.33)	49.26, 45.75 (59.4, 51.58)	12.18, 9.11 (16.45, 9.11)	11.87, 8.81 (16.2, 8.92)	5.32, 3.78 (6.91, 2.55)	2.25, 1.49 (3.43, 0.89)
	MAX-MCUSUM	135.03, 126.13 (145.63, 132.93)	17.95, 15.03 (25.99, 18.42)	11.35, 9.01 (18.03, 11.29)	4.95, 3.8 (7.88, 3.42)	2.33, 1.69 (3.92, 1.19)	1.25, 0.51 (2.21, 0.44)
	SS-EWMAe	118.4, 104.8 (129.91, 114.94)	42.25, 30.94 (45.83, 31.58)	17.07, 9.11 (16.26, 6.36)	16.9, 8.88 (16.23, 6.31)	8.83, 3.89 (8.56, 2.41)	3.47, 1.71 (4.22, 0.96)
	SS-CUSUMe	142.03, 132.15 (147.66, 133.7)	42.7, 35.49 (47.37, 35.52)	11.64, 7.8 (13.83, 5.97)	11.91, 8.02 (14.06, 6.11)	5.74, 3.53 (7.14, 2.2)	2.4, 1.4 (3.61, 0.88)
1.3	MAX-MEWMA	12.14, 10.28 (19.32, 13.66)	10.8, 8.96 (17.32, 11.45)	7.03, 5.43 (10.94, 5.56)	7.22, 5.5 (11.1, 5.66)	4.05, 3.04 (5.93, 2.20)	2.08, 1.36 (3.27, 0.88)
	MAX-MCUSUM	57.59, 52.95 (73.54, 65.29)	13.81, 11.21 (21.73, 15.53)	8.53, 6.85 (14.63, 9.22)	4.69, 3.5 (7.68, 3.39)	2.3, 1.64 (3.9, 1.23)	1.27, 0.57 (2.23, 0.46)
	SS-EWMAe	23.95, 16.3 (35.89, 24.52)	19.03, 11.41 (25.04, 14.06)	12.28, 6.52 (13.23, 4.98)	12.12, 6.29 (13.11, 4.83)	7.47, 3.69 (7.69, 2.3)	3.2, 1.67 (4.04, 0.96)
	SS-CUSUMe	65.35, 59.6 (81.19, 71.57)	27.45, 22.95 (35.52, 26.67)	9.49, 6.52 (12.57, 5.5)	9.5, 6.63 (12.54, 5.68)	4.71, 3.25 (6.47, 2.13)	2.17, 1.34 (3.41, 0.87)
2	MAX-MEWMA	2.58, 1.77 (4.79, 1.78)	2.55, 1.79 (4.74, 1.77)	2.46, 1.66 (4.53, 1.55)	2.45, 1.71 (4.46, 1.56)	2.17, 1.45 (3.84, 1.2)	1.58, 0.93 (2.69, 0.77)
	MAX-MCUSUM	5.34, 4.54 (9.47, 6.53)	4.28, 3.43 (8.11, 5.17)	3.54, 2.78 (6.45, 3.63)	3.02, 2.25 (5.57, 2.67)	2.04, 1.43 (3.52, 1.23)	1.33, 0.67 (2.24, 0.49)
	SS-EWMAe	5.22, 2.78 (7.47, 2.52)	5.15, 2.77 (7.29, 2.4)	4.82, 2.6 (6.57, 2.12)	4.88, 2.65 (6.6, 2.1)	3.89, 2.2 (5.11, 1.51)	2.3, 1.35 (3.4, 0.85)
	SS-CUSUMe	4, 3.16 (8.06, 4.18)	3.79, 2.86 (7.59, 3.55)	3.32, 2.44 (6.31, 2.56)	3.37, 2.47 (6.39, 2.63)	2.6, 1.86 (4.6, 1.55)	1.72, 1.06 (2.92, 0.79)

**Table 3:** ATS=ARL, SDTS=SDRL for FP and ATS, SDTS (ARL, SDRL) for VP schemes for shifts in the second slope vector (${{\varvec{\beta}}}_{2}$) and the error variation ($\tau$), when *p*=2 and *q*=2.

	***Shift in*** ${\varvec \beta }_{2}=\left({\beta }_{21},{\beta }_{22}\right)$.						
$\tau$	Control chart	(0, 0)	(0.05, 0.05)	(0.1, 0)	(0.1, 0.1)	(0.2, 0.2)	(1, 1)
FP scheme							
1	MAX-MEWMA	200, 200	176.07, 179.05	99.65, 97.07	99.9, 97.43	22.18, 16.59	4.32, 1.31
	MAX-MCUSUM	200, 195	48.24, 38.8	47.99, 39.43	20.32, 13.32	8.68, 3.76	3.26, 0.83
	SS-EWMAe	200, 190	103.07, 94.25	38.56, 30.78	37.35, 29.6	11.85, 5.78	4.17, 0.99
	SS-CUSUMe	200, 200	101.41, 94.79	36.37, 30.04	37.97, 30.98	11.95, 6.18	4.22, 0.92
1.1	MAX-MEWMA	108.71, 105.42	93.99, 91.65	59.3, 52.93	57.84, 53.42	18.44, 13.08	1.93, 0.47
	MAX-MCUSUM	159.94, 152.48	44.69, 37.38	44.76, 36.36	20.03, 12.93	8.61, 3.81	1.88, 0.35
	SS-EWMAe	143, 134.89	76.56, 69.1	32.34, 27.02	33.13, 26.01	11.63, 6.06	2.1, 0.37
	SS-CUSUMe	148.21, 145.14	82.87, 74.61	33.72, 28.06	34.18, 27.6	11.49, 5.91	2.07, 0.47
1.3	MAX-MEWMA	26.19, 22.23	25.36, 20.76	21.73, 17.37	21.21, 17.33	12.35, 8.4	1.88, 0.52
	MAX-MCUSUM	106.76, 100.12	37.73, 30.83	37.41, 29.5	19.1, 12.85	8.59, 4.14	1.85, 0.39
	SS-EWMAe	50.95, 45.33	38.08, 32	22.97, 17	23.1, 16.75	10.34, 5.42	2.1, 0.39
	SS-CUSUMe	89.39, 81.7	57.96, 52.3	28.95, 23.12	28.18, 21.95	11.18, 5.81	2.04, 0.51
2	MAX-MEWMA	5.18, 2.68	5.14, 2.6	5.05, 2.53	5.11, 2.6	4.61, 2.23	1.69, 0.56
	MAX-MCUSUM	14.3, 9.32	13.05, 8.37	12.46, 7.82	10.77, 6.35	7.34, 3.75	1.82, 0.48
	SS-EWMAe	7.91, 4.12	7.81, 3.93	7.3, 3.64	7.34, 3.61	5.99, 2.7	1.99, 0.45
	SS-CUSUMe	14.17, 10.84	13.56, 9.72	11.69, 7.98	11.61, 7.95	7.99, 4.45	1.89, 0.58
VP scheme							
1	MAX-MEWMA	200, 205(200, 205)	162.32, 167.2(167.28, 168.78)	62.75, 59.03(72.41, 65.51)	63.63, 58.97(72.64, 65.1)	12.2, 7.73(16.44, 8.23)	3.59, 1.99(4.97, 1.34)
	MAX-MCUSUM	200, 200(200, 195)	37.02, 30.18(49.47, 38.3)	35.86, 29.7(48.68, 38.12)	12.68, 9.46(20.61, 13.33)	4.14, 2.95(7.88, 3.39)	1.36, 0.56(3.05, 0.66)
	SS-EWMAe	200, 180(200, 180)	87.14, 75.58(88.98, 76.48)	33.35, 20.63(34.35, 20.24)	32.59, 19.95(33.66, 19.56)	14.79, 6.08(14.29, 4.96)	5.03, 1.95(5.62, 1.3)
	SS-CUSUMe	200, 180(200, 180)	83.76, 74.21(86.74, 73.3)	28.46, 20.79(32.18, 20.54)	28.79, 21.17(32.49, 20.61)	10.88, 6.04(13.08, 4.92)	3.37, 1.7(5.16, 1.19)
1.1	MAX-MEWMA	82, 81.93(92.22, 88.33)	64.15, 63.51(74.71, 70.89)	34.4, 31.03(43.6, 36.33)	34.12, 30.79(43.54, 36.69)	10.41, 6.99(14.74, 7.48)	1.2, 0.38(2.29, 0.68)
	MAX-MCUSUM	135.03, 126.13(145.63, 132.93)	31.4, 25.19(43.22, 32.5)	29.66, 24.54(42.21, 32.34)	11.65, 8.66(19.24, 12.31)	4.12, 2.95(7.81, 3.49)	1.09, 0.02(1.93, 0.26)
	SS-EWMAe	118.4, 104.8(129.91, 114.94)	61.04, 50.78(67.35, 54.42)	28.04, 17.19(30.37, 17.68)	28.79, 17.47(30.99, 17.88)	13.74, 5.94(13.63, 4.72)	1.36, 0.56(2.78, 0.54)
	SS-CUSUMe	142.03, 132.15(147.66, 133.7)	67.39, 59(72.87, 60.66)	25.9, 18.71(30.37, 19.33)	26.24, 19.08(30.54, 19.22)	10.06, 5.66(12.64, 4.69)	1.2, 0.34(2.42, 0.59)
1.3	MAX-MEWMA	12.14, 10.28(19.32, 13.66)	11.22, 9.51(17.9, 12.41)	9.79, 8.05(15.83, 10.13)	9.63, 7.94(15.55, 10.33)	6.43, 4.66(10.38, 5.06)	1.19, 0.38(2.16, 0.69)
	MAX-MCUSUM	57.59, 52.95(73.54, 65.29)	21.11, 17.48(32.54, 24.96)	19.16, 16.44(30.66, 24.53)	9.69, 7.27(16.89, 10.93)	3.85, 2.82(7.51, 3.47)	1.09, 0.041(1.92, 0.28)
	SS-EWMAe	23.95, 16.3(35.89, 24.52)	20.7, 12.68(29.3, 18.063)	16.16, 9.06(20.46, 10.39)	15.87, 8.82(20.21, 10.23)	10.6, 4.89(11.65, 4.07)	1.34, 0.55(2.68, 0.59)
	SS-CUSUMe	65.35, 59.6(81.19, 71.57)	39.17, 33.13(50, 39.9)	19.44, 14.21(25.76, 16.19)	19.06, 13.98(25.67, 15.94)	8.32, 5.15(11.68, 4.77)	1.19, 0.34(2.32, 0.61)
2	MAX-MEWMA	2.58, 1.77(4.79, 1.78)	2.55, 1.75(4.76, 1.74)	2.56, 1.77(4.73, 1.71)	2.45, 1.66(4.64, 1.66)	2.39, 1.61(4.4, 1.49)	1.14, 0.34(1.86, 0.69)
	MAX-MCUSUM	5.34, 4.54(9.47, 6.53)	4.49, 3.62(8.6, 5.56)	4.35, 3.48(8.13, 5.18)	3.95, 3.17(7.65, 4.6)	2.71, 1.93(5.47, 2.64)	1.09, 0.11(1.86, 0.37)
	SS-EWMAe	5.22, 2.78(7.47, 2.52)	5.13, 2.83(7.33, 2.53)	4.96, 2.67(7.07, 2.35)	5.18, 2.76(7.18, 2.38)	4.51, 2.38(6.27, 1.94)	1.28, 0.51(2.42, 0.68)
	SS-CUSUMe	4, 3.16(8.06, 4.18)	3.94, 3.02(7.99, 3.85)	3.79, 2.79(7.53, 3.53)	3.74, 2.84(7.43, 3.52)	3.2, 2.32(6.22, 2.52)	1.15, 0.31(2.08, 0.63)

The results in all these three tables show that in all the FP and VP control charts, as the intercept/slope or variability shift increases, the charts signal faster. Moreover, if the number of profiles whose intercepts/slopes shift increases from one to two, i.e. from (0.2, 0) to (0.2, 0.2) in Table 1, only the Max-MCUSUM chart shows a significant increase in the performance (decrease in the ATS value), and the performance of the other charts remains more or less the same. In addition, by comparing the charts’ FP and VP schemes, we realize that all the charts show significant performance improvements if the VP scheme is used (with more than a 70% performance increase in some cases), and by comparing different control charts, it is clear that the Max-type control charts mostly perform better than the SS-type control charts (only if one profile shifts, the SS-type control charts perform better and that also only in some cases of no or low variability shifts). As for the Max-type control charts, the Max-MEWMA chart mostly performs better as the variability shift increases and the Max-MCUSUM chart mostly performs better as the mean shift increases.

For the case of *p*=6, the real case adopted in Sabahno & Amiri^27^ with the following model is used:

$${y}_{1}=-0.05+10{x}_{1}-0.01{x}_{2}-0.03{x}_{3}+0.26{x}_{4}+0{x}_{5}+0.03{x}_{6}+{\varepsilon }_{1},$$

$${y}_{2}=0.48+0.24{x}_{1}+21.01{x}_{2}-0.09{x}_{3}+0.03{x}_{4}-0.12{x}_{5}+0.01{x}_{6}+{\varepsilon }_{2},$$

$${y}_{3}=0.37+0.09{x}_{1}+0.01{x}_{2}+6.81{x}_{3}+0.04{x}_{4}+0.02{x}_{5}-0.03{x}_{6}+{\varepsilon }_{3},$$

$${y}_{4}=0.04+0{x}_{1}+0{x}_{2}+0{x}_{3}+10.53{x}_{4}-0.47{x}_{5}+0.21{x}_{6}+{\varepsilon }_{4},$$

$${y}_{5}=0.09-0.021{x}_{1}+0{x}_{2}+0.01{x}_{3}+0.02{x}_{4}+7{x}_{5}-0.34{x}_{6}+{\varepsilon }_{5},$$

$${y}_{6}=0.09+0.04{x}_{1}+0{x}_{2}-0.01{x}_{3}+0.18{x}_{4}-0.34{x}_{5}+11.46{x}_{6}+{\varepsilon }_{6}.$$

Since there are two sampling strategies in our adaptive scheme, we use ${n}_{1}=8$ and ${n}_{2}=16$, and $E\left(n\right)=12$. Therefore, since we have two sets of sample sizes, we need two value sets for the explanatory variables as well. Again, we use the same value sets used by Sabahno & Amiri^27^, which we don’t include in this paper to save space.

The results of this case are presented in Tables 4, 5 and 6, for separate and simultaneous shifts in the variability and the intercepts, separate and simultaneous shifts in the variability and the first slopes, and separate and simultaneous shifts in the variability and the second slopes, respectively.

**Table 4:** ATS=ARL, SDTS=SDRL for FP and ATS, SDTS (ARL, SDRL) for VP schemes for shifts in the intercept vector (${{\varvec{\beta}}}_{0}$) and the error variation ($\tau$), when *p*=6 and *q*=6.

	***Shift in*** ${\varvec \beta }_{0}=\left({\beta }_{01},{\beta }_{02},...,{\beta }_{06}\right)$.						
$\tau$	Control chart	(0,.., 0)	(0.1,.., 0.1)	(0.2, 0,..., 0)	(0.2,..., 0.2)	(0.5,…, 0.5)	(1,…, 1)
FP scheme							
1	MAX-MEWMA	200, 200	198.7, 209.82	189.91, 208.1	196.25, 210.76	190.14, 195.74	201.01, 209.45
	MAX-MCUSUM	200, 195	212.9, 210.27	204.62, 196.48	222.39, 218.41	245.19, 230.01	344.87, 347.44
	SS-EWMAe	200, 190	196.26, 185.62	192.67, 185.84	194.02, 187.84	167.71, 165.28	87.43, 81.78
	SS-CUSUMe	200, 200	199.83, 192.25	187.01, 186.06	194.18, 188.72	161.62, 152.3	88.12, 81.36
1.1	MAX-MEWMA	52.41, 50.68	53.74, 52.87	51.94, 50.26	51.92, 50.65	50.79, 49.25	49.82, 47.42
	MAX-MCUSUM	106.25, 106.57	105.78, 101.91	103.86, 100.43	114.33, 111.36	118.02, 114.85	132.01, 128.21
	SS-EWMAe	79.43, 72.4	80.01, 70.26	76.93, 70.92	76.07, 69	71.46, 61.13	47.98, 39.77
	SS-CUSUMe	140.41, 136.78	141.71, 140.18	136.61, 128.99	135.7, 127.08	111.59, 110.47	66.34, 60.13
1.3	MAX-MEWMA	6.72, 4.63	6.55, 4.61	6.63, 4.68	6.7, 4.8	6.52, 4.6	6.51, 4.53
	MAX-MCUSUM	11.59, 7.95	11.41, 7.98	11.4, 7.9	11.62, 8.17	11.07, 7.96	11.25, 7.73
	SS-EWMAe	11.29, 6.22	11.41, 6.1	11.25, 5.91	11.4, 6.24	10.94, 5.88	10.73, 5.72
	SS-CUSUMe	32.58, 26.75	32.87, 26.28	32.54, 27.23	31.83, 26.04	31.73, 25.85	25.2, 18.74
2	MAX-MEWMA	1.5, 0.59	1.47, 0.57	1.46, 0.58	1.49, 0.61	1.46, 0.59	1.46, 0.58
	MAX-MCUSUM	2.07, 0.73	2.05, 0.74	2.05, 0.72	2.06, 0.73	2.07, 0.73	2.02, 0.7
	SS-EWMAe	2.64, 0.71	2.63, 0.7	2.62, 0.68	2.66, 0.69	2.63, 0.68	2.62, 0.68
	SS-CUSUMe	2.73, 0.89	2.69, 0.85	2.68, 0.85	2.66, 0.85	2.65, 0.82	2.66, 0.85
VP scheme							
1	MAX-MEWMA	200, 220 (200, 220)	192.27, 216.33 (191.95, 213.77)	192.64, 213.75 (192.67, 211.55)	200.23, 216.32 (200.09, 214.78)	197.94, 220.07 (198.21, 218.28)	191.63, 208.64 (192.78, 207.18)
	MAX-MCUSUM	200, 185 (200, 185)	196.78, 195.27 (195.03, 191.37)	203.53, 200.71 (203.81, 197.33)	211.34, 200.76 (209.04, 196.11)	243.15, 241.55 (237.17, 232.53)	291.31, 297.39 (277.41, 280.02)
	SS-EWMAe	200, 190 (200, 190)	202.05, 189.51 (202.67, 186.45)	197.88, 193 (198.82, 190.08)	186.08, 180.82 (186.82, 176.56)	150.29, 140.32 (153.22, 136.78)	66.35, 53.37 (70.27, 53.34)
	SS-CUSUMe	200, 200 (200, 200)	202.18, 207.68 (202.18, 200.11)	193.65, 203.87 (194.69, 196.8)	192.76, 196.34 (193.3, 189.04)	146.54, 140.94 (149.93, 136.46)	63.83, 57.74 (69.68, 56.69)
1.1	MAX-MEWMA	25.89, 27.64 (35.5, 35.05)	24.85, 25.63 (34.17, 32.37)	26.3, 26.3 (35.51, 32.79)	25.61, 25.78 (35.14, 32.89)	24.57, 25.25 (33.71, 32)	22.3, 22.37 (31.26, 29.11)
	MAX-MCUSUM	63.2, 62.19 (82.07, 78.58)	63.73, 60.05 (82.23, 74.55)	64.71, 62.97 (84.1, 79.78)	64.76, 60.38 (83.34, 75.54)	70.64, 69.05 (89.28, 84.56)	69.51, 70.32 (87.58, 85.92)
	SS-EWMAe	34.05, 27.77 (54.82, 43.52)	33.05, 26.36 (52.07, 40.18)	31.9, 24.62 (50.61, 38.11)	32.27, 27.03 (53.4, 42.11)	28.88, 21.45 (45.07, 31.81)	23.07, 15.62 (33.79, 21.15)
	SS-CUSUMe	122.73, 120.67 (141.29, 132.13)	119.31, 112.98 (137.93, 124.94)	115.48, 114.29 (132.96, 124.96)	114.3, 113.67 (132.06, 124.32)	86.34, 81.8 (102.48, 90.76)	41.25, 35.42 (52.38, 39.17)
1.3	MAX-MEWMA	2.2, 1.64 (4.88, 2.4)	2.17, 1.52 (4.83, 2.28)	2.19, 1.64 (4.85, 2.36)	2.24, 1.66 (4.89, 2.33)	2.22, 1.65 (4.78, 2.41)	2.22, 1.67 (4.86, 2.35)
	MAX-MCUSUM	4.42, 3.83 (9.52, 6.95)	4.5, 3.93 (9.63, 6.84)	4.63, 3.85 (9.68, 6.69)	4.35, 3.6 (9.53, 6.53)	4.51, 3.74 (9.53, 6.65)	4.47, 3.72 (9.43, 6.8)
	SS-EWMAe	5.56, 2.85 (9.05, 3.01)	5.67, 2.94 (9.04, 3.05)	5.63, 2.97 (8.95, 2.89)	5.54, 2.86 (8.93, 2.96)	5.62, 2.95 (9.08, 2.97)	5.54, 2.84 (8.88, 2.89)
	SS-CUSUMe	4.74, 4.41 (12.42, 6.83)	4.54, 4.1 (12.47, 7.24)	4.48, 3.91 (12.07, 6.59)	4.67, 4.05 (12.63, 7.06)	4.39, 3.85 (11.88, 6.49)	4.59, 4.08 (11.79, 6.28)
2	MAX-MEWMA	1.06, 0.18 (1.51, 0.56)	1.05, 0.16 (1.47, 0.56)	1.07, 0.2 (1.5, 0.56)	1.06, 0.17 (1.48, 0.55)	1.06, 0.19 (1.5, 0.57)	1.07, 0.2 (1.5, 0.56)
	MAX-MCUSUM	1.13, 0.28 (1.99, 0.56)	1.13, 0.28 (2.01, 0.57)	1.14, 0.29 (1.97, 0.58)	1.14, 0.3 (2, 0.59)	1.14, 0.3 (1.97, 0.56)	1.14, 0.31 (1.99, 0.59)
	SS-EWMAe	1.42, 0.65 (3.01, 0.52)	1.43, 0.63 (3.01, 0.52)	1.43, 0.64 (3, 0.54)	1.42, 0.61 (3.01, 0.53)	1.43, 0.65 (3.02, 0.52)	1.43, 0.65 (2.99, 0.53)
	SS-CUSUMe	1.2, 0.29 (2.66, 0.57)	1.21, 0.3 (2.69, 0.56)	1.2, 0.28 (2.7, 0.56)	1.2, 0.26 (2.67, 0.56)	1.2, 0.28 (2.68, 0.58)	1.19, 0.24 (2.67, 0.56)

**Table 5:** ATS=ARL, SDTS=SDRL for FP and ATS, SDTS (ARL, SDRL) for VP schemes for shifts in the first slope vector (${{\varvec{\beta}}}_{1}$) and the error variation ($\tau$), when *p*=6 and *q*=6.

	***Shift in*** ${\varvec \beta }_{1}=\left({\beta }_{11},{\beta }_{12},...,{\beta }_{16}\right)$						
$\tau$	Control chart	(0,.., 0)	(0.02,.., 0.02)	(0.05, 0,..., 0)	(0.05,..., 0.05)	(0.1, …, 0.1)	(0.2, …, 0.2)
FP scheme							
1	MAX-MEWMA	200, 200	183.99, 199.62	109.4, 111.28	105.5, 106.2	14.55, 11.18	2.7, 0.89
	MAX-MCUSUM	200, 195	33.65, 28.5	85.39, 81.8	8.7, 5.24	3.4, 1.27	1.71, 0.5
	SS-EWMAe	200, 190	196.65, 201.05	158.36, 156.68	166.79, 157.38	64.11, 54.94	6.56, 2.66
	SS-CUSUMe	200, 200	193.81, 193.56	166.35, 163	164.36, 168.25	109.92, 108.61	11.5, 6.7
1.1	MAX-MEWMA	52.41, 50.68	47.41, 45.84	30.25, 27.76	28.5, 26.5	8.57, 6.08	2.44, 0.86
	MAX-MCUSUM	106.25, 106.57	29.68, 24.8	50.59, 47.21	8.26, 4.95	3.43, 1.32	1.73, 0.52
	SS-EWMAe	79.43, 72.4	72.76, 66.04	55.95, 49.06	54.42, 48.39	21.9, 15.33	5.05, 1.79
	SS-CUSUMe	140.41, 136.78	133.1, 129.57	118.49, 110.63	119.68, 115.57	69.77, 60.86	7.17, 3.37
1.3	MAX-MEWMA	6.72, 4.63	6.6, 4.73	5.86, 4.04	5.65, 3.79	3.87, 2.15	1.89, 0.72
	MAX-MCUSUM	11.59, 7.95	9.81, 6.43	9.12, 5.75	6.1, 3.39	3.26, 1.31	1.71, 0.54
	SS-EWMAe	11.29, 6.22	10.99, 5.83	9.65, 4.94	9.78, 5.04	7.12, 3.1	3.5, 0.98
	SS-CUSUMe	32.58, 26.75	31.72, 25.67	25.37, 20.48	24.63, 19.23	12.98, 8.21	4.05, 1.53
2	MAX-MEWMA	1.5, 0.59	1.46, 0.58	1.44, 0.58	1.45, 0.58	1.34, 0.52	1.15, 0.37
	MAX-MCUSUM	2.07, 0.73	2.02, 0.73	1.99, 0.7	1.98, 0.68	1.81, 0.61	1.37, 0.49
	SS-EWMAe	2.64, 0.71	2.63, 0.68	2.59, 0.67	2.57, 0.65	2.45, 0.6	2.06, 0.41
	SS-CUSUMe	2.73, 0.89	2.68, 0.86	2.6, 0.82	2.64, 0.81	2.44, 0.73	1.95, 0.54
VP scheme							
1	MAX-MEWMA	200, 220 (200, 220)	182.94, 201.13 (184.08, 199.41)	54.85, 53.17 (63.35, 59.32)	53.81, 56.13 (62.35, 62.57)	4.71, 3.25 (7.65, 3.49)	1.64, 0.89 (2.91, 0.72)
	MAX-MCUSUM	200, 185 (200, 185)	55.19, 49.22 (67.95, 59.9)	88.45, 82.75 (106.36, 98.49)	14.73, 9.39 (23.22, 15.5)	3.97, 2.38 (10.2, 6.72)	1.12, 0.13 (2.18, 0.86)
	SS-EWMAe	200, 190 (200, 190)	192.5, 184.75 (193.85, 182.43)	141.78, 136.54 (149.85, 142.13)	143.7, 139.44 (152.49, 142.76)	25.06, 18.17 (39.8, 26.84)	3.39, 1.85 (5.93, 1.64)
	SS-CUSUMe	200, 200 (200, 200)	193.05, 196.23 (193.4, 189.23)	155.75, 156.31 (158.54, 151.08)	154.83, 156.47 (158.36, 152.7)	67.44, 63.75 (81.38, 70.13)	2.01, 1.39 (6.13, 2.14)
1.1	MAX-MEWMA	25.89, 27.64 (35.5, 35.05)	22.8, 23.56 (31.98, 30.12)	10.67, 10 (17.21, 13.82)	10.54, 9.89 (16.98, 13.94)	3.03, 2.26 (5.71, 2.38)	1.43, 0.73 (2.58, 0.76)
	MAX-MCUSUM	63.2, 62.19 (82.07, 78.58)	29.55, 25.82 (45.35, 38.55)	23.25, 21.62 (37.22, 33.55)	10.14, 7.64 (20.38, 15.38)	2.57, 1.6 (7.09, 4.01)	1.1, 0.13 (1.99, 0.75)
	SS-EWMAe	34.05, 27.77 (54.82, 43.52)	30.56, 23.08 (49.35, 36.02)	19.18, 13.51 (33.55, 22.11)	19.76, 13.83 (33.81, 22.13)	8.49, 4.96 (14.36, 6.2)	2.62, 1.44 (4.83, 1.24)
	SS-CUSUMe	122.73, 120.67 (141.29, 132.13)	118.73, 119.2 (138.76, 131.78)	79.98, 75.66 (101.7, 89.85)	83.14, 78.12 (105.01, 92.08)	18.63, 17.53 (36.22, 27.58)	1.62, 0.85 (4.56, 1.35)
1.3	MAX-MEWMA	2.2, 1.64 (4.88, 2.4)	2.18, 1.59 (4.75, 2.2)	1.94, 1.38 (4.3, 1.97)	2, 1.36 (4.34, 1.89)	1.65, 0.97 (3.38, 1.23)	1.17, 0.38 (2.03, 0.73)
	MAX-MCUSUM	4.42, 3.83 (9.52, 6.95)	3.93, 3.22 (8.95, 6.16)	3.12, 2.51 (6.8, 4.4)	2.72, 1.99 (6.79, 4.24)	1.58, 0.84 (3.89, 1.8)	1.08, 0.11 (1.81, 0.58)
	SS-EWMAe	5.56, 2.85 (9.05, 3.01)	5.42, 2.8 (8.71, 2.75)	5.04, 2.59 (8.09, 2.53)	5.06, 2.6 (8.14, 2.55)	3.84, 1.99 (6.34, 1.79)	1.86, 1.02 (3.66, 0.78)
	SS-CUSUMe	4.74, 4.41 (12.42, 6.83)	4.49, 4.16 (11.86, 6.74)	3.71, 3.13 (10.09, 5.26)	3.65, 3.15 (10.17, 5.15)	2.33, 1.68 (6.52, 2.48)	1.33, 0.5 (3.33, 0.71)
2	MAX-MEWMA	1.06, 0.18 (1.51, 0.56)	1.05, 0.19 (1.47, 0.55)	1.05, 0.14 (1.46, 0.54)	1.05, 0.13 (1.46, 0.55)	1.04, 0.15 (1.37, 0.51)	1.01, 0.071 (1.15, 0.36)
	MAX-MCUSUM	1.13, 0.28 (1.99, 0.56)	1.12, 0.24 (1.98, 0.55)	1.13, 0.29 (1.94, 0.55)	1.12, 0.23 (1.95, 0.55)	1.09, 0.16 (1.81, 0.51)	1.04, 0.04 (1.42, 0.49)
	SS-EWMAe	1.42, 0.65 (3.01, 0.52)	1.42, 0.63 (3, 0.5)	1.41, 0.62 (2.98, 0.52)	1.39, 0.59 (2.97, 0.51)	1.34, 0.54 (2.84, 0.52)	1.16, 0.25 (2.34, 0.57)
	SS-CUSUMe	1.2, 0.29 (2.66, 0.57)	1.19, 0.26 (2.67, 0.58)	1.19, 0.25 (2.64, 0.57)	1.18, 0.22 (2.63, 0.56)	1.17, 0.21 (2.49, 0.56)	1.11, 0.09 (2.06, 0.52)

**Table 6:** ATS=ARL, SDTS=SDRL for FP and ATS, SDTS (ARL, SDRL) for VP schemes for shifts in the second slope vector (${{\varvec{\beta}}}_{2}$) and the error variation ($\tau$), when *p*=6 and *q*=6.

	***Shift in*** ${\varvec \beta }_{2}=\left({\beta }_{21},{\beta }_{22},...,{\beta }_{26}\right)$						
$\tau$	Control chart	(0,.., 0)	(0.05,.., 0.05)	(0.1, 0,..., 0)	(0.1,..., 0.1)	(0.2, …, 0.2)	(1, …, 1)
FP scheme							
1	MAX-MEWMA	200, 200	200.3, 209.01	170.8, 173.78	173.62, 179.37	68.9, 70.27	1.37, 0.48
	MAX-MCUSUM	200, 195	242.54, 243.19	209.27, 203.62	298.75, 300.15	386.36, 385.9	1.75, 0.53
	SS-EWMAe	200, 190	208.63, 208.66	187.3, 177.75	181.68, 169.56	134.9, 125.82	2.51, 0.6
	SS-CUSUMe	200, 200	194.29, 189.69	187.73, 186.07	186.84, 182.78	147.99, 139.26	2.54, 0.69
1.1	MAX-MEWMA	52.41, 50.68	49.22, 48.82	39.28, 37.91	41.05, 40.06	21.37, 19	1.28, 0.45
	MAX-MCUSUM	106.25, 106.57	114.6, 112.92	97.79, 96.7	120.25, 113.07	78.36, 76.98	1.64, 0.55
	SS-EWMAe	79.43, 72.4	79.77, 70.82	68.09, 59.28	69.63, 61.41	44.19, 36.94	2.35, 0.52
	SS-CUSUMe	140.41, 136.78	138.98, 133.04	132.54, 125.77	131.12, 126.44	99.87, 94.57	2.33, 0.61
1.3	MAX-MEWMA	6.72, 4.63	6.6, 4.89	6.22, 4.42	6.32, 4.37	5.32, 3.6	1.15, 0.36
	MAX-MCUSUM	11.59, 7.95	11.23, 7.89	10.58, 7.43	10.37, 7.11	8.52, 5.38	1.43, 0.51
	SS-EWMAe	11.29, 6.22	11.07, 6.22	10.73, 5.69	10.79, 5.9	9.06, 4.53	2.1, 0.41
	SS-CUSUMe	32.58, 26.75	32.2, 27.2	30.02, 24.28	29.52, 23.28	21.57, 16.11	2.03, 0.51
2	MAX-MEWMA	1.5, 0.59	1.47, 0.59	1.46, 0.59	1.43, 0.57	1.41, 0.55	1.01, 0.12
	MAX-MCUSUM	2.07, 0.73	2.04, 0.7	2, 0.72	2.04, 0.72	1.94, 0.66	1.09, 0.29
	SS-EWMAe	2.64, 0.71	2.65, 0.69	2.64, 0.69	2.63, 0.664	2.58, 0.66	1.63, 0.49
	SS-CUSUMe	2.73, 0.89	2.68, 0.84	2.64, 0.83	2.66, 0.87	2.57, 0.82	1.4, 0.49
VP scheme							
1	MAX-MEWMA	200, 220 (200, 220)	186.6, 208.69 (187.91, 207.79)	138.04, 146.15 (143.22, 149.32)	139.52, 151.44 (143.99, 154.09)	20.95, 19.69 (28.02, 24.61)	1.05, 0.15 (1.41, 0.5)
	MAX-MCUSUM	200, 185 (200, 185)	178.28, 176.02 (180.83, 174.48)	176.04, 167.89 (183.6, 173.13)	147.22, 142.12 (153.26, 144.95)	59.7, 56.39 (71.71, 64.1)	1.08, 0.13 (1.77, 0.42)
	SS-EWMAe	200, 190 (200, 190)	193.97, 190.46 (195.15, 187.73)	181.5, 180.51 (183.86, 179.64)	178.87, 167.22 (182.17, 167.09)	103.02, 96.09 (117.21, 106.09)	1.27, 0.4 (2.88, 0.43)
	SS-CUSUMe	200, 200 (200, 200)	190.95, 204.73 (191.6, 195.02)	173.59, 168.9 (175.59, 163.07)	189.28, 192.12 (190.61, 186.71)	135.92, 136.48 (141.04, 134.66)	1.16, 0.11 2.59, 0.52
1.1	MAX-MEWMA	25.89, 27.64 (35.5, 35.05)	23.11, 24.01 (32.13, 30.39)	17.72, 17.18 (25.97, 22.87)	18.08, 18.4 (26.18, 23.49)	7.01, 6.14 (12.05, 8.36)	1.03, 0.09 (1.31, 0.46)
	MAX-MCUSUM	63.2, 62.19 (82.07, 78.58)	55.17, 52.53 (72.3, 66.92)	45.47, 43.85 (62.43, 57.44)	40.62, 39.34 (56.56, 52.16)	14.33, 13.71 (23.47, 20.27)	1.07, 0.12 (1.68, 0.46)
	SS-EWMAe	34.05, 27.77 (54.82, 43.52)	32.67, 25.32 (52.73, 40.38)	25.87, 20 (43.73, 32.8)	26.64, 19.37 (44.93, 32.27)	14.78, 9.41 (26.63, 15.61)	1.24, 0.37 (2.76, 0.48)
	SS-CUSUMe	122.73, 120.67 (141.29, 132.13)	113.62, 112.21 (132.43, 124.38)	105.25, 104.66 (125.12, 116.78)	104.8, 100.98 (124.88, 114.99)	65.21, 59.74 (88.79, 75.18)	1.14, 0.13 (2.4, 0.53)
1.3	MAX-MEWMA	2.2, 1.64 (4.88, 2.4)	2.24, 1.61 (4.88, 2.34)	2.08, 1.42 (4.57, 2.07)	2.07, 1.49 (4.63, 2.21)	1.92, 1.24 (4.13, 1.71)	1.01, 0.05 (1.18, 0.38)
	MAX-MCUSUM	4.42, 3.83 (9.52, 6.95)	4.22, 3.52 (8.96, 6.25)	3.86, 3.14 (8.32, 5.55)	3.75, 3.1 (8.16, 5.5)	2.79, 2.14 (5.85, 3.33)	1.05, 0.06 (1.49, 0.5)
	SS-EWMAe	5.56, 2.85 (9.05, 3.01)	5.66, 2.76 (9.06, 2.91)	5.32, 2.73 (8.56, 2.77)	, 5.33, 2.75 (8.52, 2.69)	4.7, 2.42 (7.63, 2.29)	1.16, 0.21 (2.45, 0.53)
	SS-CUSUMe	4.74, 4.41 (12.42, 6.83)	4.53, 3.94 (12.23, 6.67)	4.13, 3.66 (11.18, 6.09)	4.12, 3.61 (11.11, 5.95)	3.29, 2.66 (9.14, 4.39)	1.11, 0.04 (2.13, 0.49)
2	MAX-MEWMA	1.06, 0.18 (1.51, 0.56)	1.06, 0.17 (1.49, 0.55)	1.06, 0.17 (1.49, 0.56)	1.06, 0.19 (1.49, 0.56)	1.06, 0.2 (1.46, 0.55)	1, 0.01 (1.01, 0.12)
	MAX-MCUSUM	1.13, 0.28 (1.99, 0.56)	1.13, 0.29 (1.99, 0.55)	1.12, 0.23 (1.97, 0.56)	1.14, 0.3 (2, 0.56)	1.12, 0.27 (1.92, 0.54)	1.01, 0.03 (1.12, 0.33)
	SS-EWMAe	1.42, 0.65 (3.01, 0.52)	1.43, 0.63 (3.01, 0.51)	1.4, 0.61 (2.99, 0.51)	1.41, 0.63 (2.99, 0.53)	1.41, 0.63 (2.96, 0.53)	1.07, 0.09 (1.72, 0.49)
	SS-CUSUMe	1.2, 0.29 (2.66, 0.57)	1.21, 0.31 (2.66, 0.58)	1.21, 0.29 (2.68, 0.59)	1.2, 0.28 (2.68, 0.58)	1.19, 0.24 (2.61, 0.58)	1.05, 0.05 (1.51, 0.5)

The results in the *p*=6 problem show that while in the cases of slope shifts (Tables 5 and 6) the conclusions are almost the same as in the previous case (*p* = 2), the same does not completely apply to the case of the intercept shift (Table 4). The Max-type control charts’ performance mostly gets worse (or their performance remains rather unchanged) as the shift in the intercept increases (with the Max-MCUSUM chart being the worst between the two). On the contrary, The SS-type charts’ performance mostly gets better (or their performance remains rather unchanged) under a similar situation. In addition, except for some cases of no/low variation shifts, the Max-MEWMA control charts perform better than the other control charts. Moreover, the VP adaptive control charts are still mostly much faster than the FP charts.

By comparing the *p *= 6 case with the *p *= 2 case, we realize that in the case of no variation shift ($\tau$ = 1), all the charts perform worse when the mean shift is in the intercept. However, if the mean shift is in the slopes, and also in the case of low variation shift ($\tau$ = 1.1) when there is no mean (intercept/slops) shift, all the charts perform better, but as the mean shift increases, the charts mostly tend to perform worse when the process dimension increases. Furthermore, in the cases of moderate/large variation shifts ($\tau$ = 1.3 and 2), all the charts perform better in the case of* p*=6 compared to the case of *p *= 2.

## A real case

To illustrate how one can implement the proposed control charts in practice, we study a real healthcare-related case. Stroke is one of the most common causes of death and disability in the world. In addition to severe consequences for individuals, stroke causes a high financial burden on societies. Intravenous thrombolysis within 4.5 hours from the stroke onset is an established treatment for ischemic stroke. The benefit of treatment reduces for every minute’s delay (Darehed et al.^40^), and thrombolysis delay times are key quality indicators of stroke care, and essential to monitor and maintain a good quality of stroke care.

We study a dataset containing all the stroke patients who received thrombolysis in Sweden from 2016 until the year 2020 and were registered in the national quality register for stroke care in Sweden (Riksstroke; Asplund et al.^41^). The study has been performed in accordance with the relevant guidelines/regulations. The Declaration of Helsinki was followed. Patients were informed about registration in Riksstroke, that their registered data could be used for research purposes, and their right to remove themselves from the registry at any time (opt-out consent). According to the Swedish Patient Data Act, data from national quality registers may be processed for research purposes without additional individual consent, if processing has been approved by an Ethics Review Board in accordance with the Ethical Review Act. The use of data from Riksstroke for this study was approved by the Swedish Ethical Review Authority (reference no. 2021-06152-01).

The objective of this study is to monitor the efficiency of the stroke care process in terms of thrombolysis treatment delays. Therefore, we investigate whether the relationship between two correlated responses, *y*_1_ = the time from stroke onset until getting the treatment (onset-to-needle time, ONT) and *y*_2_ = the time from admission to the hospital until getting the treatment (door-to-needle time, DNT) in relation to three crucial covariates (patient characteristics) of* x*_*1*=_Age, *x*_2=_Sex, and *x*_3=_Stroke Severity (as measured by NIH stroke score, i.e. NIHSS) is being kept constant over time or not. *x*_*1*_ and* x*_*3*_ are modeled as continuous variables and *x*_2_ as a binary variable (0 for male and 1 for female).

We used the data from two recent years 2016 and 2017 (which were considered to be stable years in the stroke system), from all the hospitals in Sweden to estimate this relationship (profile).

After cleaning the dataset by removing the missing data (the proportion of missing data was relatively low compared to the overall dataset) and the erroneous entries (times more than 4.5 hours, which showed they had been entered wrongly), our first analysis was to see if ONT and DNT were normally distributed or not. The analysis revealed that their distributions were skewed, indicating that they deviated from a normal distribution and appeared more like Lognormal distributions. This outcome is commonly expected for time-related variables with many factors influencing them. Consequently, we applied a logarithmic transformation (base 10) to the data in order to approximate a normal distribution. The variance-covariance matrix of these response variables was estimated as: $$\Sigma =\left[\begin{array}{cc}0.0399& 0.0207\\ 0.0207& 0.0743\end{array}\right]$$.

Then, we estimated the multivariate multiple regression model using R. The results were as follows.

$${\text{log}}\left({y}_{1}\right)=2.0457+0.00084{x}_{1}+ 0.01597{x}_{2}-0.0045{x}_{3},$$

$${\text{log}}\left({y}_{2}\right)=1.6797-0.00079{x}_{1}+0.01558{x}_{2}-0.0032{x}_{3}.$$

We used the VP adaptive control charts (Max-MEWMA, Max-MCUSUM, SS-EWMAe, and SS-CUSUMe) to monitor these regression models over time. The design parameters for our control charts are the same as in our simulation study section (except for the sampling intervals) are set to ${n}_{1}=4$, ${n}_{2}=8$, $E\left(n\right)=6$, $E\left(\alpha \right)=0.005$, ${\alpha }_{1}=0.004$, $E\left(t\right)=2$ *months* and*,* ${t}_{2}=1$ *month.* The other chart parameters are computed using Equations (23)-(25) and the proposed algorithms in Sect. "Design parameters in a variable parameters scheme" and can be seen in Tables 7, 8, 9 and 10 for each control chart.

**Table 7:** Details of process monitoring from Jan 2018 to the end of 2020 in the real case for the Max-MEWMA control chart.

*k*	${n}_{k}$	∑*n*_k_	*t*_k_	∑*t*_k_	$\overline{y}_{k1}$.	$\overline{y}_{k2}$	*C*_*i*_	*S*_*i*_	*ME*_*i*_	$UW{L}_{k}$	$UC{L}_{k}$	Status
1	4	4	0	0	2.026	1.6126	− 2.370	− 0.544	2.3709	1.015	3.02	In-control
2	8	12	1	1	1.9795	1.3841	− 1.3818	0.4899	1.3818	1.027	2.88	In-control
3	8	20	1	2	2.0025	1.6123	− 0.9026	0.3047	0.9026	1.027	2.88	In-control
4	4	24	3	5	2.1742	1.5832	− 1.6976	− 0.5666	1.6976	1.015	3.02	In-control
5	8	32	1	6	2.1556	1.5356	− 0.8181	− 0.4981	0.8181	1.027	2.88	In-control
6	4	36	3	9	2.2056	1.5118	0.5411	0.0312	0.5411	1.015	3.02	In-control
7	4	40	3	12	2.1488	1.8032	− 0.372	− 0.1467	0.3720	1.015	3.02	In-control
8	4	44	3	15	2.034	1.5701	0.7043	0.0895	0.7043	1.015	3.02	In-control
9	4	48	3	18	1.8667	1.439	2.654	1.9659	2.654	1.015	3.02	In-control
10	8	56	1	19	1.9888	1.4648	0.5034	− 0.6222	0.6222	1.027	2.88	In-control
11	4	60	3	22	2.0069		10.53	1.7444	**10.53**	1.015	**3.02**	**Out-of-control**
12	8	68	1	23	2.1511	1.6276	6.0024	− 0.39284	**6.0024**	1.027	**2.88**	**Out-of-control**

Significant values are in bold.

**Table 8:** Details of process monitoring from Jan 2018 to the end of 2020 in the real case for the Max-MCUSUM control chart.

*k*	${n}_{k}$	∑*n*_k_	*t*_k_	∑*t*_k_	$\overline{y}_{k1}$.	$\overline{y}_{k2}$	*U*_*i*_	*L*_*i*_	*MC*_*i*_	*UWL*_*k*_	*UCL*_*k*_	Status
1	4	4	0	0	2.026	1.6126	1.8698	0	1.8698	4	21.1	In-control
2	4	8	3	3	2.011	1.3841	0.7745	0	0.7745	4	21.1	In-control
3	4	12	3	6	2.0992	1.5923	0	0	0	4	21.1	In-control
4	4	16	3	9	2.2056	1.5118	9.455	0	9.455	4	21.1	In-control
5	8	24	1	10	2.0926	1.6137	8.4685	0	0	1.25	8.5	In-control
6	4	28	3	13	1.7671	1.548	6.533843	0.6072	6.5338	4	21.1	In-control
7	8	36	1	14	2.205	1.5931	0	0	0	1.25	8.5	In-control
8	4	40	3	17	2.1538	1.5321	0	0	0	4	21.1	In-control
9	4	44	3	20	2.2077	1.4511	21.3979	0	**21.3979**	**4**	**21.1**	**Out-of-control**
10	8	52	1	21	2.1674	1.394	0	0.7704	0.7704	1.25	8.5	In-control
11	4	56	3	24	2.0492	1.5049	28.7707	0	**28.7707**	**4**	**21.1**	**Out-of-control**

Significant values are in bold.

**Table 9:** Details of process monitoring from Jan 2018 to the end of 2020 in the real case for the SS-EWMAe control chart.

*k*	${n}_{k}$	∑*n*_k_	*t*_k_	∑*t*_k_	$\overline{y}_{k1}$	$\overline{y}_{k2}$	*P*_*i*_	*V*_*i*_	*EWe*_*i*_	$UW{L}_{k}$	$UC{L}_{k}$	Status
1	4	4	0	0	2.026	1.6126	− 0.3005	− 0.18152	0.12330	0.252	2.04	In-control
2	4	8	3	3	2.011	1.567	− 0.4886	− 0.4779	0.4671	0.252	2.04	In-control
3	8	16	1	4	2.1213	1.7809	− 0.3756	− 0.4298	0.3258	0.2521	1.8359	In-control
4	8	24	1	5	2.1245	1.6451	− 0.3074	− 0.1069	0.1059	0.2521	1.8359	In-control
5	4	28	3	8	2.115	1.611	− 0.4153	− 0.1285	0.189	0.252	2.04	In-control
6	4	32	3	11	2.1121	1.3769	− 0.3941	0.302	0.2466	0.252	2.04	In-control
7	4	36	3	14	2.3107	1.6291	− 0.0781	0.5422	0.3001	0.252	2.04	In-control
8	8	44	1	15	2.0615	1.5852	0.2096	0.5602	0.3578	0.2521	1.8359	In-control
9	8	52	1	16	2.0718	1.5599	0.4177	0.7012	0.6662	0.2521	1.8359	In-control
10	8	60	1	17	2.0818	1.5522	0.5384	0.1824	0.3232	0.2521	1.8359	In-control
11	8	68	1	18	2.0011	1.3946	0.7016	0.6836	0.9596	0.2521	1.8359	In-control
12	8	76	1	19	2.1128	1.5361	0.8698	0.6515	1.1811	0.2521	1.8359	In-control
13	8	84	1	20	2.0701	1.5125	1.0042	0.7787	1.6149	0.2521	1.8359	In-control
14	8	92	1	21	2.1674	1.394	1.4368	0.9083	**2.8897**	**0.2521**	**1.8359**	**Out-of-control**
15	8	100	1	22	2.1047	1.5665	1.715	0.9593	**3.8616**	**0.2521**	**1.8359**	**Out-of-control**
16	8	108	1	23	2.1511	1.6276	1.8704	0.8024	**4.1426**	**0.2521**	**1.8359**	**Out-of-control**

Significant values are in bold.

**Table 10:** Details of process monitoring from Jan 2018 to the end of 2020 in the real case for the SS-CUSUMe control chart.

*k*	n_k_	∑*n*_k_	*t*_k_	∑*t*_k_	$\overline{y}_{k1}$	$\overline{y}_{k2}$	*M*_*i*_	*N*_*i*_	*CUe*_*i*_	$UW{L}_{k}$	$UC{L}_{k}$	Status
1	4	4	0	0	2.026	1.6126	0.5029	0	0.2529	0.0043	23	In-control
2	8	12	1	1	1.9805	1.5844	0	0	0	0.003	16	In-control
3	4	16	3	4	2.1689	1.4409	0	0	0	0.0043	23	In-control
4	4	20	3	7	2.1764	1.6922	0	0	0	0.0043	23	In-control
5	4	24	3	10	2.0327	1.6042	0.9359	0	0.876	0.0043	23	In-control
6	8	32	1	11	1.9531	1.4321	0.1228	0.3603	0.1449	0.003	16	In-control
7	8	40	1	12	2.0403	1.6441	0	0	0	0.003	16	In-control
8	4	44	3	15	2.034	1.5701	0	0	0	0.0043	23	In-control
9	4	48	3	18	1.882	1.2385	0.3239	1.5975	2.657	0.0043	23	In-control
10	8	56	1	19	2.1128	1.5361	0.8717	0.6011	1.1213	0.003	16	In-control
11	8	64	1	20	2.0701	1.5125	1.3873	0.389	2.0762	0.003	16	In-control
12	8	72	1	21	2.1674	1.3940	3.1299	0.3157	9.896373	0.003	16	In-control
13	8	80	1	22	2.1047	1.5665	4.5538	0	**20.7379**	**0.003**	**16**	**Out-of-control**
14	8	88	1	23	2.1511	1.6276	5.5828	0	**31.1685**	**0.003**	**16**	**Out-of-control**

Significant values are in bold.

After designing the control charts, we first checked whether the process was really under control in Phase-I or not (during the years 2016 and 2017). Otherwise, the estimated regression models and consequently the developed control charts would not be valid to be used in Phase -II. Researchers usually use non-adaptive (FP) schemes to do so. The result showed that the process was in-control according to all the control charts (the details of this analysis are not included in this paper but can be requested from the corresponding author).

**Figure 1:**
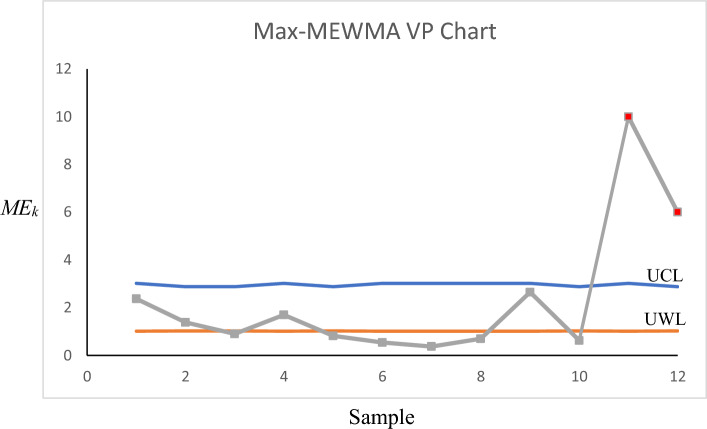
Max-MEWMA VP control chart in the illustrative example.

**Figure 2:**
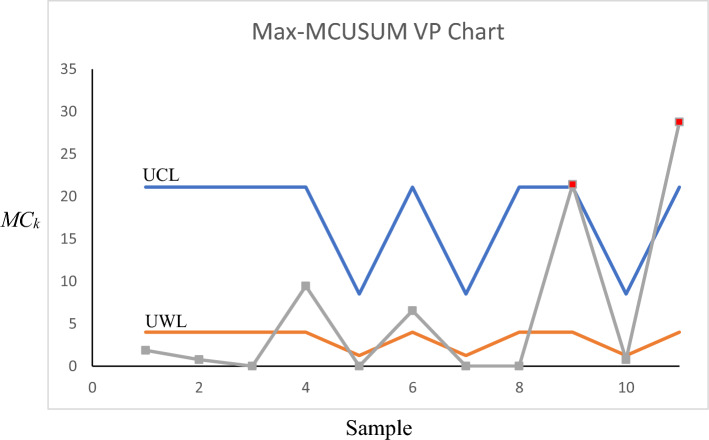
Max-MCUSUM VP control chart in the illustrative example.

**Figure 3:**
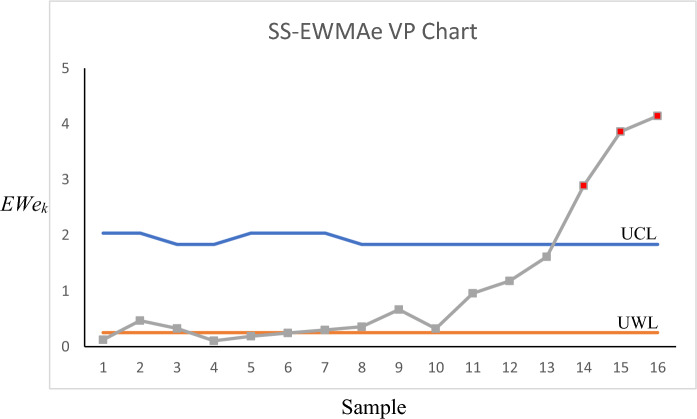
SS-EWMAe VP control chart in the illustrative example.

**Figure 4:**
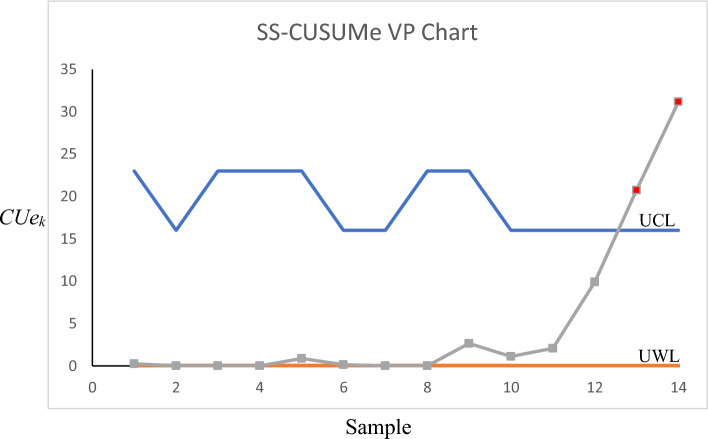
Max-CUSUMe VP control chart in the illustrative example.

For Phase-II, we employed the developed control charts for the years 2018 and 2019 to see if any assignable causes could be detected in those years. The control charts for the Max-MEWMA, Max-MCUSUM, SS-EWMAe, and SS-CUSUMe schemes can be seen in Figs. 1, 2, 3 and 4, respectively. More details regarding each sample can be seen in Tables 7, 8, 9 and 10.

Tables 7, 8, 9 and 10, from left to right, show the sample number (*k*), the sample size (the number of patients investigated in the sample), the cumulative number of samples up to the current sample, the sampling interval (in months) used to reach the current sample, the cumulative number of the sampling intervals up to the current sample (the time from the start of the process monitoring up to the current sample), the mean of the current sample's first and second response variables, the values of the sample statistics for the mean and variability, the value of the final statistic, the *UWL* and *UCL* values used for the current sample, and the status of the process based on the current sample, respectively. Remember that each control chart has two statistics, one for monitoring the mean vector and the other one for monitoring the variability. However, by using a Max or SS operator, a single final statistic will be formed and plotted in each control chart, i.e. the statistic in column ten.

As can be seen in Figs. 1, 2, 3 and 4 as well as Tables 7, 8, 9 and 10, the Max-MEWMA control chart was able to detect the out-of-control situations at samples eleventh (November 2019) and twelfth (December 2019). This control chart was able to signal first after 22 months and after observing 60 patients. The Max-MCUSUM control chart was able to detect the out-of-control situations in samples ninth (September 2019) and 11th (December 2019). This control chart was able to signal first after 20 months and after observing 44 patients. The SS-EWMAe control chart signaled at samples 14th (October 2019), 15th (November 2019), and 16th (December 2019). This chart was able to first signal after 21 months and after investigating 90 patients. Finally, the SS-CUSUMe control chart signaled at samples 13th (November 2019) and 14th (December 2019). This chart was able to first signal after 22 months and after investigating 80 patients.

One thing that is clear based on the obtained result is that in all the control charts, the statistic responsible for the mean vector monitoring (column eight) has caused the signal, meaning the shift has happened in the coefficient values and not the responses’ variability.

Further investigation is required to discover the reasons for these signals and to see which ones are real assignable causes and which ones are outliers that can be ignored. Then, these assignable causes should be removed if they are undesirable. We might even need to update the profile’s parameters if we discover that those assignable causes are desirable and should be kept.

## Conclusions

In this paper, we improved the performance of four memory-type control charts for monitoring multivariate multiple linear profiles. These control charts are Max-MEWMA, and Max-MCUSUM control charts that use a single Max-type statistic and monitor the regression parameters, and SS-EWMAe and Max-CUSUMe control charts that use an SS-type statistic and monitor the residuals. We designed a VP adaptive scheme for all these control charts, in which all the design parameters can be varied throughout the process monitoring to increase their capability in detecting shifts. After that, we developed an algorithm with which the time to signal and run length-based performance measures of these charts could be measured. Then, we performed extensive simulations to evaluate these charts’ performance under different shift sizes and types as well as different process dimensions. Two different cases of two profiles (*p* = 2)-two covariates (*q* = 2), and also, six profiles (*p* = 6)-six covariates (*q* = 6) were studied in this paper.

The results in the *p* = 2 and *q* = 2 case showed that: (i) as the intercept/slope or variability shift increases, all the FP and VP charts signal faster, (ii) if the number of profiles whose intercepts/slopes shift increases, only the Max-MCUSUM chart shows a significant performance improvement, (iii) all the charts show significant performance improvements if the VP scheme is utilized, (iv) the Max-type control charts mostly perform better than the SS-type control, and (v) the Max-MEWMA chart mostly performs better as the variability shift increases and the Max-MCUSUM chart mostly performs better as the mean shift increases. The results in the *p* = 6 and *q* = 6 case show that, in the case of slope shifts, the conclusions are more or less the same as in the case of *p* = 2 and *q* = 2. However, in the case of intercept shift, the main difference is that the Max-type control charts’ performance mostly gets worse (or their performance remains rather unchanged) as the shift in the intercept increases (with the Max-MCUSUM chart being the worst between the two) and the SS-type charts’ performance mostly gets better (or their performance remain rather unchanged).

Finally, we used a real dataset to estimate two profiles in a stroke care process and then developed and utilized the VP control charts to monitor those profiles over time to show how these charts can be implemented in real practice.

For future studies, implementing similar adaptive strategies for more advanced profiles such as non-parametric and nonlinear profiles can be suggested. Furthermore, developing and implementing other control charts to improve the healthcare-related processes in general, and the stroke care process in particular, could be a great contribution considering the availability of very few studies in this regard.

## Data Availability

The data that supports the findings of this study are available from Riksstroke, but restrictions apply to the availability of these data, which were used under license for the current study, and so are not publicly available. Data are however available from Marie Eriksson (marie.eriksson@umu.se) upon reasonable request and with the permission of Riksstroke.
